# Traits and risk factors of post-disaster infectious disease outbreaks: a systematic review

**DOI:** 10.1038/s41598-021-85146-0

**Published:** 2021-03-10

**Authors:** Gina E. C. Charnley, Ilan Kelman, Katy A. M. Gaythorpe, Kris A. Murray

**Affiliations:** 1 Department of Infectious Disease Epidemiology, School of Public Health, Imperial College London, London, UK; 2 MRC Centre for Global Infectious Disease Analysis, Imperial College London, London, UK; 3 Institute for Risk and Disaster Reduction, Faculty of Mathematical and Physical Sciences, University College London, London, UK; 4 Institute for Global Health, Faculty of Population Health, University College London, London, UK; 5 University of Agder, Kristiansand, Norway; 6 MRC Unit The Gambia At London, School of Hygiene and Tropical Medicine, Fajara, The Gambia

**Keywords:** Climate-change impacts, Climate-change mitigation, Natural hazards, Infectious diseases, Risk factors

## Abstract

Infectious disease outbreaks are increasingly recognised as events that exacerbate impacts or prolong recovery following disasters. Yet, our understanding of the frequency, geography, characteristics and risk factors of post-disaster disease outbreaks globally is lacking. This limits the extent to which disease outbreak risks can be prepared for, monitored and responded to following disasters. Here, we conducted a global systematic review of post-disaster outbreaks and found that outbreaks linked to conflicts and hydrological events were most frequently reported, and most often caused by bacterial and water-borne agents. Lack of adequate WASH facilities and poor housing were commonly reported risk factors. Displacement, through infrastructure damage, can lead to risk cascades for disease outbreaks; however, displacement can also be an opportunity to remove people from danger and ultimately protect health. The results shed new light on post-disaster disease outbreaks and their risks. Understanding these risk factors and cascades, could help improve future region-specific disaster risk reduction.

## Introduction

Despite improvements in sanitation and hygiene, along with vaccine discovery and mass immunisation schedules, infectious diseases still cause millions of annual deaths worldwide, especially in children under 5 years and in low-income countries. Several diseases have epidemic potential to cause public health emergencies, incurring large economic and social costs^1,2^. Across diseases, outbreaks have a variety of influences (e.g., food, water, sanitation, health systems) and can be exacerbated by complex interactions among such factors^3^. One influence for infectious disease outbreaks is disasters, with post-disaster outbreaks extensively reported^4–7^. Examples include an outbreak of norovirus in Texas after Hurricane Katrina in 2005^8^ and cutaneous leishmaniasis outbreaks during the Syrian conflict beginning in 2013^9^. Despite reports of these outbreaks, few studies have systematically reviewed or quantified such events or their associated risk factors on a global scale, even though this knowledge would help to indicate the resources and attention for outbreaks required from disaster risk reduction and response activities.

Here, in accordance with disaster research, the term disaster encompasses vulnerabilities interacting with a natural hazard (e.g., earthquakes, floods, droughts) or an armed conflict (e.g., terrorism, civil war)^10–12^. One potential adverse impact from a disaster is a disease outbreak. According to the World Health Organization, an infectious disease outbreak is an occurrence of a disease above normal expectancy^2^. The number of cases may vary according to the aetiological agent as well as the size and type of previous and existing exposure, while the geographic occurrence of some outbreaks may be further shaped by whether a pathogen is endemic or geographically restricted to a certain region or introduced from elsewhere afterwards.

A risk factor is here defined as a clear mechanism that contributed to the disease outbreak. Several risk factors can lead to post-disaster disease outbreaks, such as poor WASH (water, sanitation and hygiene) ^6^, alterations in disease vector distributions or behaviour^13^, issues with housing and shelter^14^, problems obtaining healthcare^15^ and population displacement^7,16–18^. All of these risk factors relate to and exacerbate pre-existing vulnerability, which are long-term processes and conditions that are the real cause of disasters while also increasing the probability of post-disaster outbreaks. To add further complexity, few risks act alone and are potentially linked, a concept known as risk factor cascades^19^.

The intersection of disasters and disease also provides an opportunity to understand the mechanisms through which global change (such as climate change) can yield health impacts^20,21^. Global change has the potential to alter some hazard parameters (e.g., intensity or frequency)^22,23^. For example, sea level rise and warming temperatures are projected to change hurricane frequency and intensity^22,24^, while altered drought frequency and intensity may influence armed conflict escalation^25,26^. These changes may be complex; for example, several studies contest the climate’s influence on conflicts^27,28^. It is therefore important to analyse and aim to estimate how communities may be impacted, in this instance by post-disaster outbreaks, so that disaster risk reduction and response can incorporate the theory regarding the cause of potential post-disaster outbreaks while being ready to address such situations in practice.

Previous research on post-disaster disease outbreaks has for the most part resulted in the collation of individual examples over specific time scales^17^, geographic areas^29^ or focused on a certain disaster^30^, resulting in limited generalisable results. Here, we aimed to conduct the first unified and comprehensive review of the literature, to gain a global overview of post-disaster disease outbreaks and their reported risk factors with no temporal limitations. We reasoned that this approach may allow us to identify links, if any, between certain hazards, vulnerabilities, disasters, geographic regions and aetiological agents. These results will allow a greater understanding of how disease outbreaks may occur in a post-disaster setting and regions and diseases commonly involved. This will highlight areas for further research and enhance disaster risk reduction by identifying areas for prioritisation to avoid post-disaster outbreaks, while providing further evidence to tackle prevalent but long-debunked statements on post-disaster outbreaks^31^. This review is therefore applicable to a range of disciplines including epidemiology, public health, disaster risk reduction and response and climate change adaptation. The specific objectives of the review are to:

1. Provide a global overview of infectious disease outbreaks that occurred in post-disaster (disasters involving either natural hazard or armed conflict) settings, to show disaster types, geographic areas affected and outbreak aetiologies;
2. Examine the risk factors that lead to these outbreaks and how they may link to form cascades.

## Results

### Search results

**Figure 1:**
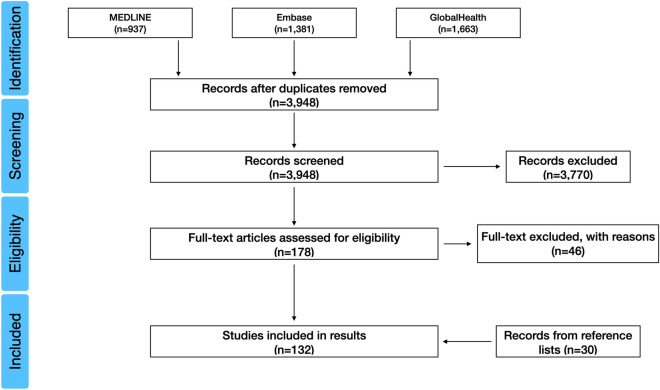
Preferred Reporting Items for Systematic Reviews and Meta-Analysis (PRISMA) diagram for the selected 132 studies on post-disaster disease outbreaks.

After screening the search results, 132 studies were selected for inclusion in the analysis (Supplementary Table 1) and a PRISMA flow diagram illustrates the selection process below (Fig. 1). Electronic database searching ceased in June 2020 but no studies after 2019 met the inclusion criteria; the studies therefore spanned from 1940 to 2019 and included ten different types of disaster and 39 different diseases across six continents. The types of studies included were retrospective and mainly involved observational studies, namely cross-sectional, case–control, case-crossover, cohort studies and epidemiological and environmental field investigations.

Several studies were either multi-disaster or multi-disease events. These were split to allow full quantification of diseases and disasters. This made 140 separate disease outbreaks and 137 separate disasters. Eight studies had only an abstract in English available, therefore the full text could not be reviewed given the caution that the searches were conducted in English only, so expansion to other languages at this stage could yield inconsistent results. While a further 25 studies were excluded because they focused on internationally displaced populations in refugee camps, while four studies with methods involving serological surveys for disease prevalence were also removed as confirmed cases of a current infection/outbreak related to a disaster could not be diagnosed by serology.

### Disaster, region and disease

Conflicts, hydrological and geophysical events were the most commonly reported disasters associated with disease outbreaks, with fewer events associated with climatological and meteorological events. A full list of reported disaster frequencies is shown in Supplementary Table 2. It is worth noting that although conflicts appear frequent (n = 45), they were not subcategorised (mainly due to the large proportion of civil wars) and are less frequently reported than all natural hazards taken together (n = 92).

**Figure 2:**
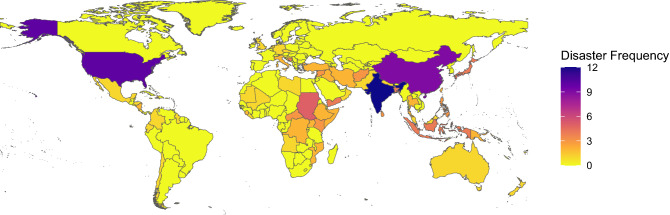
Frequency of reported post-disaster disease outbreaks by country for each 137 separate disaster events found in the literature search^32^.

**Figure 3:**
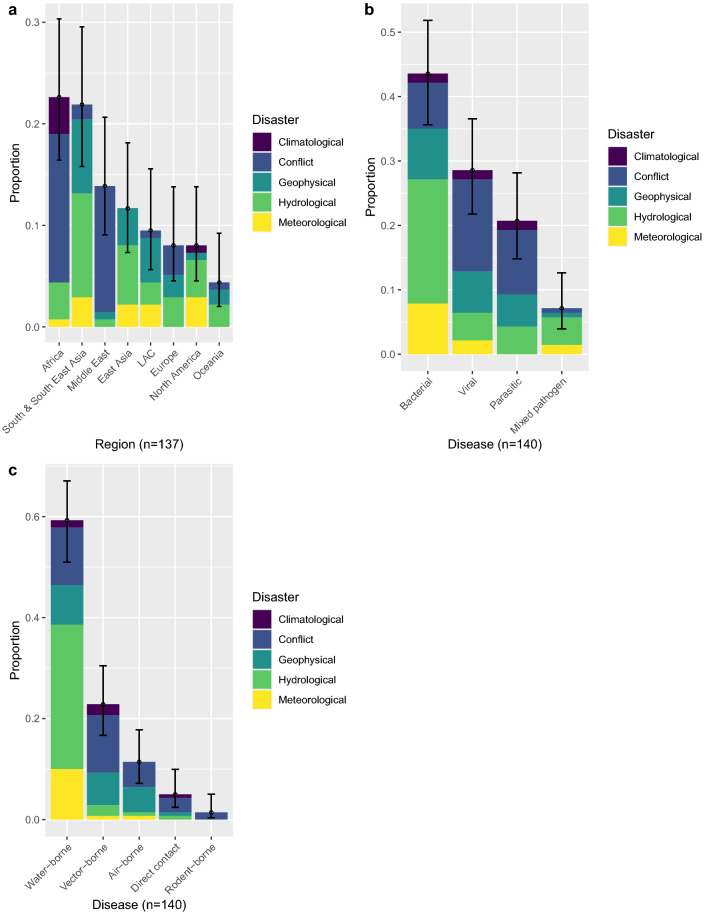
Proportion of reported post-disaster outbreaks by (**a**), region against the 137 separate disasters, (**b**), the 140 separate disease outbreaks by pathogen type against disaster and (**c**), the 140 separate disease outbreaks by transmission against disaster with binomial confidence intervals (95%). LAC – Latin America and the Caribbean.

Africa, S & SE Asia and the Middle East were strongly over-represented compared to Oceania, the Americas and Europe in post-disaster disease outbreaks (Figs. 2 and 3a). Within the regions, India (n = 12), the USA (n = 10) and China (n = 9) were predominant. A full list of reported region frequencies is shown in Supplementary Table 3 and frequency plots of Fig. 3 are shown in Supplementary Fig. 1.

**Figure 4:**
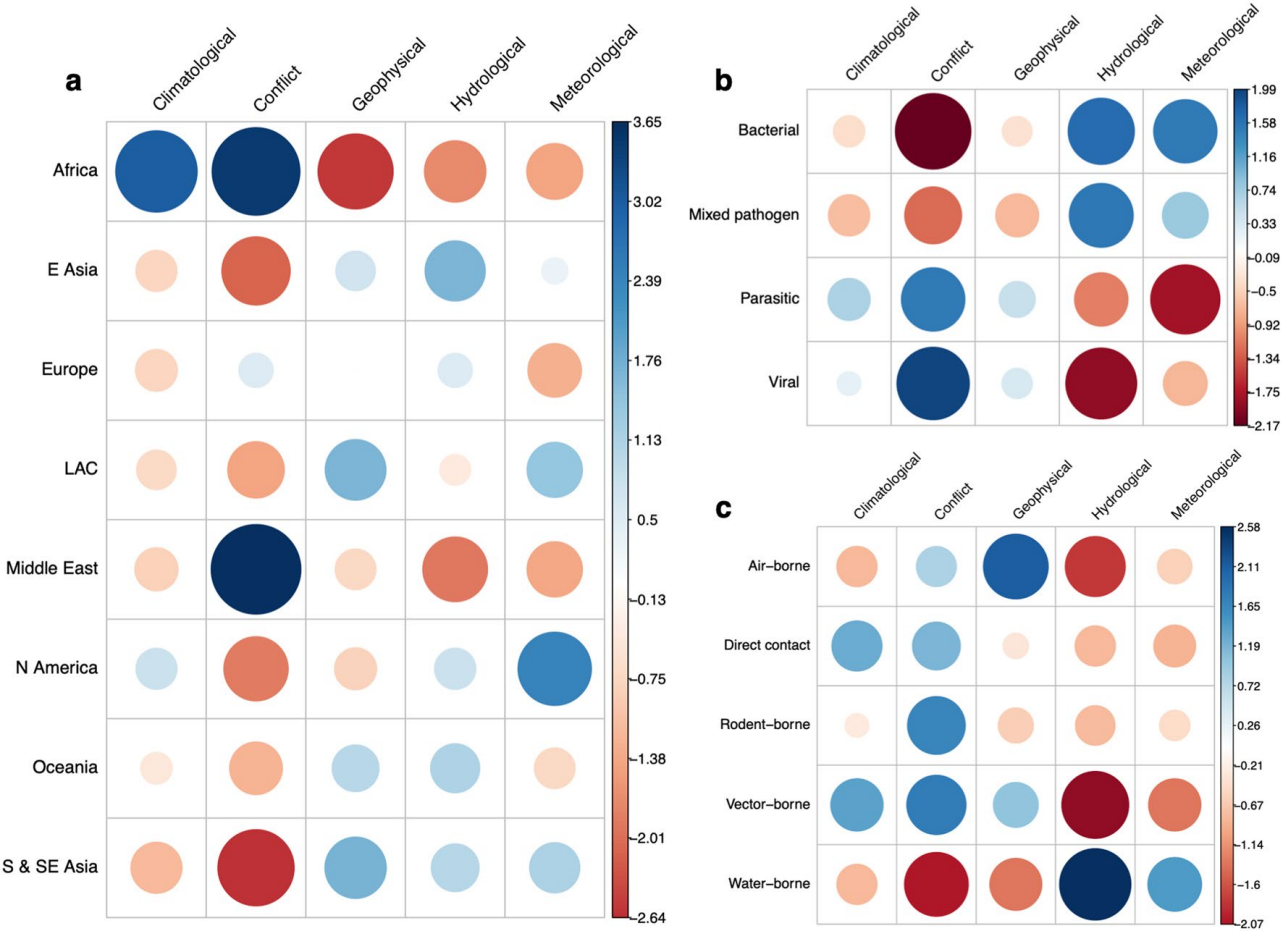
Correlation plots for the Pearson’s chi squared test residuals for each catagories in **a**, region against disaster (X-squared = 101.81, df = 28, *P*-value ≤ 0.05), **b**, disease against disaster (X-squared = 31.49, df = 12, *P*-value ≤ 0.05) and **c**, disease transmission against disaster (X-squared = 47.31, df = 16, *P*-value ≤ 0.05). Positive residuals are blue, suggesting a positive association between the corresponding row and column and negative residuals are red, suggesting a negative association.

The overrepresented regions are mainly accounted for by strong positive associations between conflict-related disease outbreaks in Africa and the Middle East (Fig. 4a), especially in Sudan and South Sudan (9/45). This contrasts with strong negative associations between African geophysical events and S & SE Asian conflicts. Africa also saw a high number of climatological-related events, reporting 5 out of 6 drought-related outbreaks (Fig. 4a). S & SE Asia mainly reported hydrological and geophysical-related outbreaks (Fig. 3a), commonly in India (12/71), along with Bangladesh and Sri Lanka (7/71).

With respect to causal agent and transmission mode of disaster-related disease outbreaks, bacterial and water-borne diseases were predominant groups (Fig. 3b,c), compared to mixed pathogen, direct contact and rodent-borne pathogens. A full list of reported aetiologies and transmission modes are shown in Supplementary Table 4.

Reported outbreaks were often disaster specific, and therefore diseases associated with hydrological events and conflicts were frequently reported. This finding is represented through strong positive correlations between bacterial or water-borne diseases and hydrological events and viral or parasitic disease and conflicts (Fig. 4b,c). This was mainly due to the number of post-flood leptospirosis (n = 18), cholera and dysentery outbreaks (n = 8). In addition, geophysical events and air-borne pathogens showed positive associations, whereas strong negative correlations were seen between conflicts and bacterial pathogens and vector-borne disease and hydrological events (Fig. 4b,c). Additional Pearson’s chi squared analysis for pair-wise comparisons is shown in Supplementary Table 5.

### Risk factors

**Figure 5:**
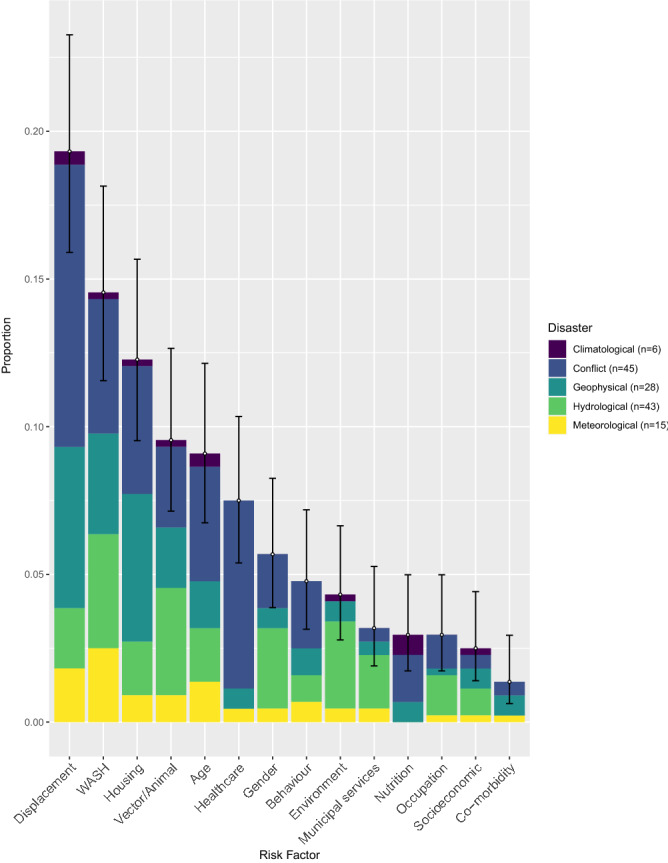
Proportions of the fourteen main risk factor clusters out of the 418 risk factors reported in the search results, against disaster, with binomial confidence intervals (95%). WASH—Water, sanitation & hygiene.

Across the 132 post-disaster disease outbreaks, 418 risk factors were reported in the studies reviewed. Individual risk factors had varying frequencies within the fourteen main clusters (Fig. 5) and how they were grouped are shown in Supplementary Table 6. Pearson’s chi-squared analysis found that risk factors were significantly different (at *P* ≤ 0.05) among post-disaster disease outbreaks. Additional figures and statistics for the fourteen main risk factor clusters against disaster, region and disease are shown in Supplementary Figs. 2–6 and in Supplementary Table 5.

**Figure 6:**
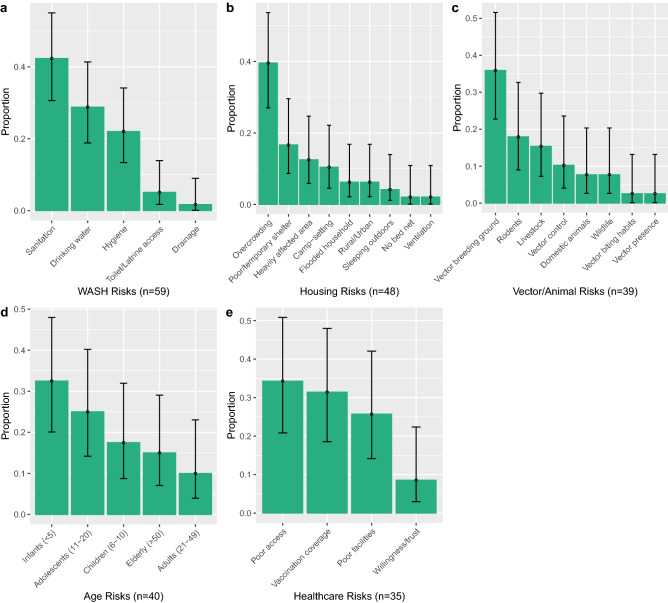
The most commonly reported risk factor clusters (**a**), WASH, (**b**), Housing (**c**), Vectors/Animals (**d**), Age and (**e**), Healthcare, split into the proportion of individual reported risk factors, with binomial confidence intervals (95%). Although displacement was the highest, it was not included as it had few elements within the cluster.

The most frequently reported risk factor was displacement, being reported 81 times, especially in relation to conflict (chi^2^ = 4.29, *P* ≤ 0.05) and geophysical events (chi^2^ = 1.51, *P* = 0.22). It was most frequently reported as a general risk factor, with no details given or to national relief camps and temporary housing. In two studies displacement was expanded upon with details on the initial and final destination e.g., rural to urban.

Water, sanitation and hygiene (WASH) was the second most commonly reported cluster (n = 59), due to poor sanitation, access to clean drinking water and poor hygiene (Fig. 6a). WASH risk factors were high in all disaster types, other than climatological, potentially due to its small sample size (n = 6). The highest frequencies of WASH risk factors were seen in hydrological events (chi^2^ = 0.3, *P* = 0.58) and conflicts (chi^2^ = 2.16, *P* = 0.14), although chi-squared analysis showed they were not significant. Instead, WASH risk factors were particularly prominent among water-borne disease outbreaks (chi^2^ = 13.64, *P* ≤ 0.05), such as leptospirosis, cholera and dysentery, and mainly attributed to the increase in standing floodwater and damage/overflow of sanitation systems.

Poor housing was the third most commonly reported cluster (n = 48), often associated with geophysical events (chi^2^ = 10.66, *P* ≤ 0.05), such as earthquakes and tsunamis. The resultant extensive infrastructure damage following these events lead to displacement in conjunction with housing risk factors, presenting through the high incidence of overcrowding (19/48), poor or temporary shelter and camp settings (13/48) (Fig. 6b).

Changes in vector (mosquito) or animal (domestic, livestock, wildlife) exposure were frequently linked with hydrological (chi^2^ = 5.17, *P* ≤ 0.05) and conflict (chi^2^ = 2.34, *P* = 0.13) events, through alterations in vector breeding ground (14/39) and vector control (4/39) (Fig. 6c), leading to parasitic diseases (chi^2^ = 8.46, *P* ≤ 0.05), such as malaria.

Of the 40 reported age-related risk factors, a quarter were in children under five years, with people under 20 years increasing that proportion to 75% (Fig. 6d). This was region and disease-specific, with several water-borne (chi^2^ = 2.13, *P* = 0.14) diarrhoea outbreaks in conflict events (chi^2^ = 0.16, *P* = 0.68) reporting children under 5 years as a risk factor.

Poor healthcare services resulting in disease outbreaks (n = 35) were particularly common in armed conflict events (chi^2^ = 30.6, *P* ≤ 0.05) compared to natural hazards. Poor access and vaccination coverage were the most common risk factors in this cluster (Fig. 6e), therefore high levels of viral diseases were reported (chi^2^ = 14.4, *P* ≤ 0.05).

Gender was reported 25 times, 5 of these stated that being female is a risk factor. A common narrative was that men assisted in post-hydrological event clean-up activities (chi^2^ = 8.5, *P* ≤ 0.05), increasing their exposure to floodwater, the most common risk factor reported in the environment cluster (13/19). This exposure enhanced their likelihood of contracting water-borne diseases (chi^2^ = 2.08, *P* = 0.15), especially leptospirosis (7/18).

### Multi-risk factor reporting and clustering

**Figure 7:**
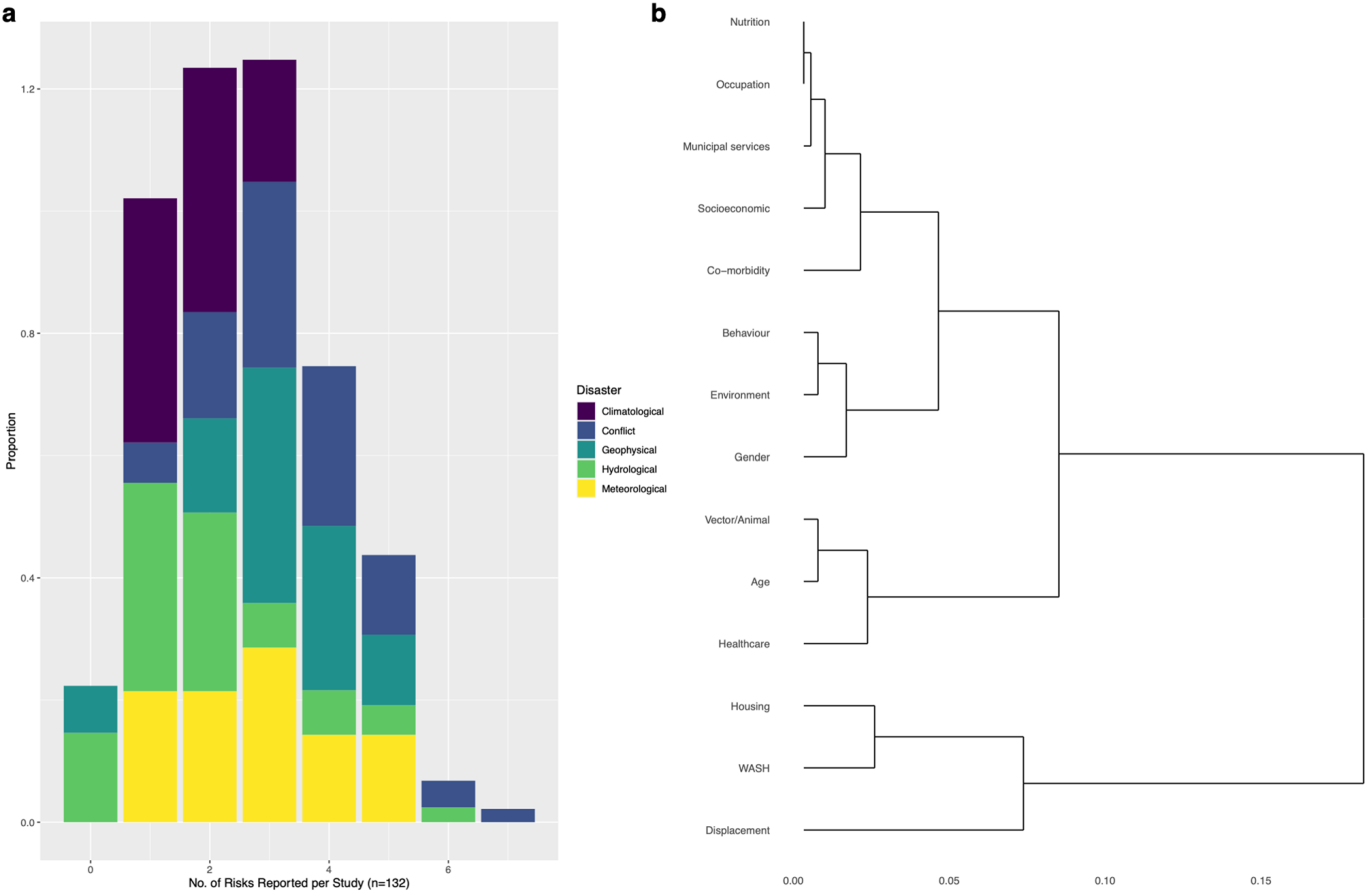
Multi-risk reporting and hierarchical clustering. (**a**), Proportions of studies (n = 132) which reported either 0 to 7 different risk factors, within the fourteen main clusters. (**b**), cluster dendrogram from hierarchical cluster analysis for the fourteen main risk factor clusters. Individual segments (leaves) on the lower part of the tree are more related to each other, as indicated by distances between the branches. The scale bar showing the dissimilarity distance between the proportions of each risk cluster.

Most of the reviewed disease outbreaks were associated with multiple risk factor clusters; almost half of studies cited two (29/132) or three (32/132) risk factors (Fig. 7a). This is also underestimated, as multiple risk factors were often reported within each cluster, for each outbreak (Supplementary Table 6). Of the comparatively few studies that reported zero (n = 8) or one (n = 22) risk factors, several (3/7 and 4/20, respectively) were in studies where only an abstract was available and therefore risk factors may have been discussed in the full text. Of the studies that reported at least 1 risk factor cluster (n = 124), conflicts were most common (n = 46) and India and China were the most common countries reporting multiple risk factors with 8 and 7 multi-risk factor outbreaks, respectively. Unspecified or multi-pathogen diarrhoeal disease and cholera were the most frequent multi-risk factor diseases, but the commonality of these groups may present the comparatively large number of reported outbreaks.

The hierarchical clustering analysis (Fig. 7b and Supplementary Fig. 7) helps to illustrate and understand the relationships between risk factors and how they were reported together. It is clear that displacement, WASH and housing were the most related risk factors here. Thirteen studies reported WASH and housing risk factor clusters together, mainly through overcrowding (n = 10), hygiene (n = 7) and sanitation (n = 6). WASH risk factors were also commonly reported with displacement, being reported together 12/13 times. Healthcare and age were reported together eleven times, eight of which were in children < 15 and mainly reported issues with vaccination coverage or poor access. The gender of these children was more commonly male. Of the twelve occurrences that age and gender were reported together, seven were in males < 20 years old. The similarity of gender, the environment and behaviour were predominantly through male exposure to floodwater and assisting in post-disaster clean-up, as previously discussed, with gender and hydrological events also showing statistical significance, as shown above.

## Discussion

The results shed new light on post-disaster disease outbreaks and their risk factors and further understanding of the frequency, geography and characteristics of these disease outbreaks globally. The most important results identified here include the large numbers of bacterial or water-borne disease due to hydrological events in South Asia and viral diseases in African conflicts. Risk factors were often disaster-specific and appeared to depend on the conditions in which the disaster occurred. This possibly explains why diseases were also disaster-specific, as certain disasters created ideal conditions for specific pathogens. The hierarchical clustering showed further evidence for the multifaceted nature of these outbreaks and the idea of risk factor cascades contributing to these outbreaks. Displacement was involved with many other risk factors, resulting in poor health outcomes and also involved in spreading diseases to new areas, as seen with Lassa fever in Sierra Leone^33^. Loss of infrastructure and the resultant displacement appears to be important in both armed conflicts and natural hazards, leading to damage to habitual residence, healthcare and services. Examples include destruction of healthcare and housing after an earthquake in Japan, leading to a pneumonia outbreak^34^ and difficulties in accessing care in Yemen during the ongoing conflict and resultant cholera outbreak^35^. Despite these conditions being potentially important in both natural hazards and armed conflicts, how they yield negative health impacts may be different and only conflict and displacement proved to be statistically significant from chi-squared analysis.

**Figure 8:**
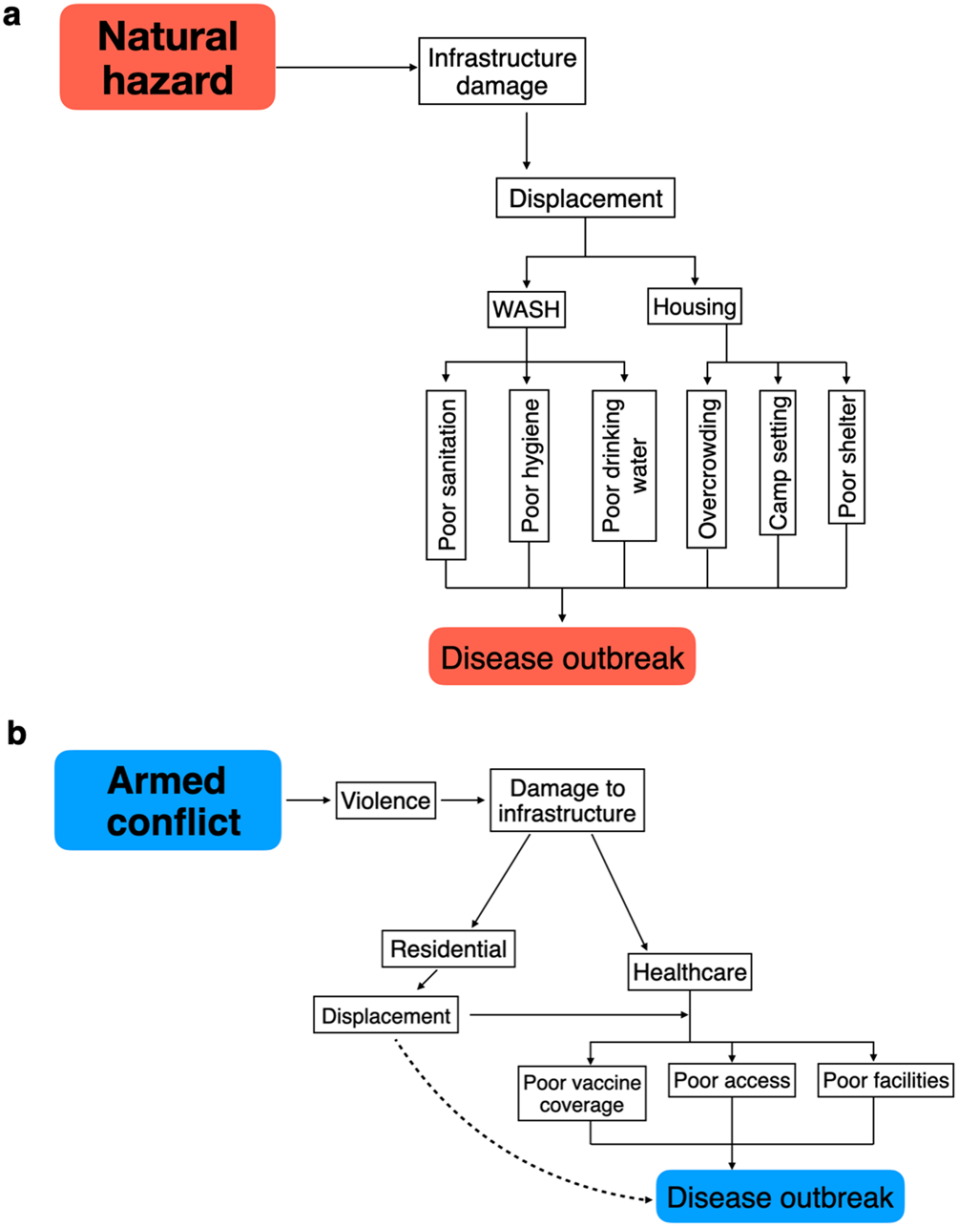
Shows an example of cascading risk factors for (**a**)**,** natural hazards and (**b**), armed conflicts. The dashed line between displacement and disease outbreaks in **8b** represents the authors understanding that displacement does not directly lead to disease outbreaks, but instead the conditions it creates.

Natural hazards may result in risk factor cascades driven by displacement (Fig. 8a), due to significant infrastructure damage. This damage can occur through flooding involving meteorological or hydrological events. Alternatively, it occurs through direct damage in geophysical events, with geophysical events and displacement showing a significant relationship. This damage and floodwater generally led to an increase in poor living conditions and an inability to maintain hygiene standards and access clean water, explained through the clustering of displacement, WASH and housing in the cluster analysis. This is presented in over half of the reported WASH risk factors, occurring in post-hydrological or post-meteorological events and the significant relationship between poor WASH conditions and water-borne diseases. Flooding also lead to increased exposure to groundwater and overflowing sewage systems. These conditions can expand vector breeding grounds, increasing the contact between populations and vectors and the resultant increase in disease cases^36,37^. In contrast to this, Fig. 4c showed a strong negative association between hydrological events and vector-borne disease. Vector breeding can be more complex than space to breed (e.g., standing water), and other factors (e.g., temperature, salinity) may prevent vectors breeding in floodwater. Floodwater is also known to destroy breeding grounds, instead of creating new ones^38^.

In armed conflict events, cascades may result from loss of infrastructure to the healthcare sector, limiting people’s access to and quality of health services (Fig. 8b), especially for children. Statistical analysis adds further evidence to this statement, finding a significant relationship between healthcare risk factors and conflict and similarity between healthcare and age in the cluster analysis. Vaccination coverage was a commonly reported risk factor in these events, potentially accounting for the significant relationship between healthcare risk factors and viral disease. Fourteen out of twenty conflict-related viral outbreaks were vaccine-preventable diseases, including hepatitis (A & E), polio and measles. Mass vaccination campaigns are commonly run through humanitarian aid organisations and as conflicts escalate, these services are often suspended due to safety concerns^15^. Another study suggested that despite high measles vaccination coverage in the Central African Republic an outbreak still occurred, due to reporting issues and poor cold-chain maintenance^39^. A factor also seen in conflicts includes healthcare forming the political fabric of the violence, resulting in attacks on health centres and workers. This further reduces uptake of services as people do not perceive seeking care as safe^40^ and mistrust can escalate towards both the government and healthcare providers^41^. For example, conflict in the Democratic Republic of Congo has reportedly hampered Ebola response teams in the 2018–2020 outbreak, dampening Ebola vaccine effectiveness to a minimum of 4.8%^42^.

Despite a common negative narrative used for displacement, one study discussed displacement as a protective factor. Reporting on West Nile virus after Hurricane Katrina, the study states that displacement allowed people to move away from floodwater and therefore vector breeding grounds^36^. A lack of displacement may be a sign of inequity and poor socioeconomic conditions, as people do not have the financial means to move and therefore become trapped within the affected area^43^. Displacement can be an opportunity to move people out of immediate danger from the disaster and provide services quickly and easily to large groups. Unfortunately, this opportunity is rarely capitalised on. For example, of the 25 times being male was reported as a risk factor, 17 of these were during outbreaks where displacement was not reported to have occurred. This is not simply a representation of the commonality of non-displacement, as displacement was reported on more occasions (n = 73) than not (n = 54). Interestingly, exposure to floodwater was also reported 16 times in outbreaks without displacement, compared to just once in studies where displacement did occur. This suggests that without displacement (especially after flooding), risk factor cascades resulted from men being more likely to assist in post-natural hazard clean up, exposing them to disease.

These results and clustering help highlight the importance of basic sanitation and hygiene, regardless of disasters, as poor WASH is linked to several infectious diseases and often associated with poverty^44,45^. For example, in Kenya, only 24.3% of the population have access to adequate sanitation, a figure which is much worse for rural communities^46^. Unfortunately, studies rarely mention these non-disaster related conditions which impact population vulnerability. Instead, risk factors are solely reported in causing the outbreak but not why they occurred or previous conditions, providing a current state and not a comprehensive view of vulnerability. This is a potentially important area of future research, especially for effective disaster planning. These poor WASH conditions also help explain why children were often implicated in these results. In India, where 28% of the reported outbreaks occurred, around 1.7 million children died before the age of 5 in 2010 alone, with diarrhoea causing 13% of this mortality^47^. Similar statistics are present throughout south Asia and Africa^48^. Another possible reason is that commonly reported diseases, including polio, measles and cholera, heavily impact young children, due to physiological (rapid onset dehydration and wasting)^48^ and social differences (poor hygiene standards)^49,50^. The gendered and age-specific risk factors found in this review, stress the need for sex and age-disaggregated post-disaster data in order to try and fully understand the impacts on disease outbreaks.

The review findings have several implications for region-specific global change. For example, under climate change scenario RCP4.5 (an intermediate scenario representing moderate emissions reductions), projections are geographically heterogenous suggesting a drier Africa and the Middle East and wetter southern Asia^51^. These changes may therefore alter the frequency/intensity of droughts and floods in the future and therefore, related disease outbreaks, in areas which already reported many post-disaster disease outbreaks. Two studies^52,53^ also reported that contact with floodwater in conjunction with higher than normal temperatures was a risk factor for developing a water-borne disease. Alterations in temperatures can impact the ways pathogens and vectors behave in the environment, yielding implications from rising global temperatures^38,54,54^. Alterations in temperature and precipitation may also occur in conjunction with population growth and urban expansion, with east and south east Asia seeing the highest rates of urbanisation^56^. Many of these are low-lying coastal cities, and liable to flooding, sea level rise^57^ and potential post-disaster disease outbreaks. This combination of both climate and population changes may therefore put more people (at higher densities) at risk for post-disaster disease outbreaks. However, urbanisation provides opportunities to meet the needs of concentrated groups of people and can be an effective low-carbon way of managing and providing services and work. Successful urban planning through building design, education and provision of healthcare, could be an effective disaster mitigation strategy for large populations and possibly reducing the risk of post-disaster disease outbreaks^58,59^.

Difficulties arise when comparing one disaster to another, as disaster severity, population risks and socioeconomic conditions of affected populations are substantially different. Thus, this is not a complete list of global post-disaster disease outbreaks and outbreaks are likely to have been missed through excluding grey literature and internationally displaced populations. If populations were displaced internationally by a disaster and an outbreak occurred, it could be argued that this was caused by the disaster. Despite this, several of the reported camps housed refugees from multiple countries, linking them to multiple disasters; therefore, this would have created issues linking the outbreak to specific disasters.

Reporting bias may suggest the high number of males reporting disease and more research is needed to understand gender biases and barriers for women accessing care in post-disaster settings. For example, women may have less access to health insurance and financing or may not be allowed to attend hospitals alone due to cultural values^60,61^. Outbreaks were also particularly common in disasters that were highly publicised. For example, of the 26 earthquake and tsunami-related outbreaks, ten were due to just two natural hazards; the 2011 Japan Earthquake and Tsunami and the 2004 Indian Ocean Tsunami. This may have introduced an over-reporting bias for certain disaster types and regions, raising questions about whether these disasters saw more disease outbreaks, or whether they were more often reported. The regions that frequently reported outbreaks are also stated as having high disaster frequencies. For example, in a 2020 review Africa and the Middle East were reported as being the most conflict-prone regions^62^, while in a 2018 review of natural hazards, 141/315 hazards were reported in Asia^63^. Despite this, figures for the number of disasters have several limitations and are dependent on what was included in the counting process.

Defining a disaster and attributing an infectious disease outbreak to this event has its difficulties, as there is no consensus on when a disaster ends and recovery begins^64^. This creates limitations in assigning and comparing risk factors. A major limitation of risk factor analysis is its subjectivity. If the authors of the reviewed studies did not clearly state their risk factors and mechanisms, this resulted in an element of subjectivity in trying to interpret results. Reported risk factors also depend on the questionnaires used during the outbreak and what was asked of participants. Several of the less frequently reported risk factors may link to more common factors but were just listed differently by the authors and resulted in high uncertainty. The magnitude of a risk factor in one event may differ from others. Confirmed and probable cases ranged from two to 379,000 across the 132 studies; therefore, how risk factors were perceived and measured in such a wide range of case numbers is likely to differ, especially statistically. Despite the studies’ limitations, with 132 separate outbreaks and 418 reported risk factors, this review is significantly larger and broader in scope than other studies exploring similar subjects^17,29,30^.

Our understanding of how global change will alter risks to populations is still relatively incomplete and has become a growing area of study, including population vulnerability to disasters. Our review provides the first comprehensive global overview of these disease outbreaks and highlighted commonly reported risk factors related to both conflicts and natural hazards. Despite displacement being suggested as an important risk factor, we suggest that displacement may help mitigate several other risks and remove people from hazardous situations, ultimately protecting their health relative to those not displaced. This is an important finding for disaster and public health literature, as this changes the narrative of many previous studies and thus supports the theory and practice of disaster risk reduction and response in terms of recognising that displacement is not inherently detrimental, but the impacts depend somewhat on how the displaced people are supported. India and several African countries had particularly high outbreak reporting rates compared to other countries. Further evidence is needed to understand why this is the case, or if it is simply a by-product of their very large geographic areas and population sizes. Certain disease aetiologies were common in specific disasters, which were often reported in specific regions. This specificity is essential for international disaster risk reduction, as humanitarian and government sector can effectively prepare for and help communities withstand the impacts of post-disaster disease outbreaks through effective region-specific mitigation, while dispelling prevalent assumptions about post-disaster outbreaks which have shown to be incorrect. This supports theoretical considerations for framing the importance of and prevention regarding post-disaster outbreaks, while indicating practical guidance and advice for operations. By further understanding the risk factors involved, outbreaks can be reduced, and this study identifies better sanitation and housing as areas for prioritisation. Hazards will inevitably strike, as they have throughout history, but it is how we as a society deal with these hazards, that result in the disaster.

## Methods

We followed the PRISMA-P 2015 checklist^65^ (Supplementary Fig. 8) for systematic reviews and were guided by the methodological approach delineated by Khan et al*.*^66^. The framework was set out to follow five stages: (1) framing the question(s), (2) identifying relevant work, (3) assessing study quality, (4) summarising the evidence and (5) interpreting the findings. As data collection for systematic reviews uses exclusively secondary data, no ethical approval was needed. The full methodological protocol used in this review underwent peer review prior to commencement of this study^67^, with the key components summarised below.

### Stage 1: Framing the research questions

After preliminary research on natural hazards and armed conflicts and their risk factors for communicable disease outbreaks, it became apparent that quantification of these contextual outbreaks and their risks was insufficient to gain a clear global understanding of the issue. Due to this deficiency, the review questions were defined as follows:

1. Which pathogens, disasters, global changes and geographic areas are commonly implicated in outbreaks in a post-disaster setting?
2. Which risk factors are important in causing post-disaster disease outbreaks and how are they potentially linked to form cascades?

### Stage 2: Identifying relevant work

The following electronic databases were searched; MEDLINE, Embase and Global Health, but grey literature was not included. Reference lists of selected papers and reviews were screened for relevant papers (snowballing) and subjected to the same screening process. Both key and medical subject heading (MeSH) terms varied depending on the database and were related to; (1) natural hazards, (2) armed conflict and (3) infectious disease outbreaks (Supplementary Table 7). No standard definitions for natural hazards, armed conflicts and disease outbreaks were set, as this may have excluded important studies, along with any specific risk factors. No temporal or geographic limits were set and no specific risk factors searched to avoid bias in the search results. Electronic database searching ceased in June 2020, so any relevant literature retrieved after this date was excluded^67^.

Along with broad terms for outbreaks, specific diseases as identified by the World Health Organization^68^ as common infectious disease outbreaks following disasters were also searched, along with commonly reported diseases identified from preliminary scoping searches. Specific pathogens include those capable of causing acute outbreaks but not causing an outbreak before the disaster. Therefore, despite evidence for contextual increases^69,70^, HIV, hepatitis B, hepatitis C and tuberculosis were not searched/included, as they often cause more chronic disease and have a wide range of social implications beyond the scope of this study. Soft tissue injuries, wound infections, inhaled fungal spores and aspiration pneumonia (tsunami lung) were also not included. Such infections would only impact those that had open wounds and/or exposure to the pathogen in the environment, and as such the patient could not transmit the pathogen through environmental contamination or direct contact making it an unlikely pathway to a widespread outbreak.

### Stage 3: Assessing study quality

After the removal of duplicates, search results were screened to assess the study quality and decide on selection against an eligibility criterion, developed through the PICOS method^71^. After consideration of published tools, the National Institute of Health quality assessment tool was used for study appraisal and thresholds set for exclusion^72^. The papers were screened by the first author and ineligible papers eliminated. All titles and abstracts that met the criteria were subjected to full-text reading.

Inclusion criteria:

- Population—Any local population/community impacted by a post-disaster disease outbreak.
- Intervention—Any investigation carried out to quantify a disease outbreak and understand the risk factors.
- Comparator—Members of the disaster-affected population who did not acquire an infection during the outbreak.
- Outcomes—The primary outcome is to understand post-disaster disease outbreaks on a global scale. The secondary outcome consists of identifying the risk factors that result in these outbreaks.
- Study type – Retrospective observational studies, namely, cross-sectional, case–control and cohort studies. Full-text or abstracts in English^67^.
  Exclusion criteria:
- Papers without an explicit link between a disaster and an outbreak.
- Outbreaks in refugees/refugee camps, foreign armed forces, aid workers and international travellers, as this review aimed to look at local outbreaks in regional populations.
- Non-English abstract and full-texts, due to linguistic constraints.
- Review papers, as only primary sources were desired for this review.
- Single case reports, as these were often not seen as representative of an outbreak in this context.
- Publications discussing general risk factors, public health, mental health and other non-communicable diseases, pathogen genetics or economic costs in a post-disaster setting, as these are beyond the scope of this review and its objectives^67^.

### Stage 4: Summarising the evidence

A predetermined data charting form was used based on preliminary reading and the objectives of the review. Extracted data included information on the publication (title, authors, date, journal), disaster type, disease, case numbers, study area, study period, identified risk factors, methodological details (study design, sample sizes, laboratory tests, statistical analysis) along with any other relevant information/data. Risk factors were recorded regardless of whether the author ran statistically analyses. To ensure all relevant data were collected, the form was reviewed by other members of the research team before implementation and the data were extracted independently by the first author^67^.

To ensure that distinctions could be made between risk factors and there was no overlap, risk factor recording was a dynamic process and the exact wording reported by the study was first entered into the data charting form and then reviewed and streamlined into categories after all the studies had been read, re-evaluating studies as needed. As this process is open for interpretation, Supplementary Table 6 shows all the individual risk factors, how they were clustered and provides a definition of the risk factor cluster to improve transparency.

### Stage 5: Interpreting the findings

Categorisation was used for ease in interpreting and presenting the results. Regions were categories based upon how the results were clustered (Africa, South & South East Asia, East Asia, Europe, Latin America and the Caribbean (LAC), North America, the Middle East, Oceania and Europe). It is acknowledged that the chosen regions were somewhat non-comparable due to differences in population sizes, environments and proximity to causative factors for hazards (e.g., fault lines for geophysical hazards). Disasters were categorised into five different groups as follows and any flooding caused by a tsunami or storm was listed under the hazard causing the flooding, not hydrological, while being aware that the vulnerabilities are still the cause of the disaster:

- Conflict—any form of reported armed conflict or violence.
- Hydrological—flooding (other than flooding caused by hurricanes, cyclones, typhoons and tropical storms)
- Geophysical—earthquakes, volcanic eruption and tsunamis
- Meteorological—hurricanes, cyclones, typhoons, tropical storms
- Climatological—droughts

Slow-onset and sudden-onset hazards were considered in this review. Diseases were also categorised into disease type (bacterial, viral, parasitic and mixed pathogen) and transmission type (water-borne, vector-borne, air-borne, direct contact and rodent-borne). Risk factors were identified by any study that specifically named them as risks or was suggested to have been involved in facilitating the outbreak (either statistically or not). Results were categorised for ease of data presentation and analysis. Risk factors were divided manually into mutually exclusive clusters identified by similarities in how they result in an outbreak and preliminary reading. This formed fourteen clusters: displacement, WASH, housing, vector/animal, age, healthcare, gender, behaviour, environment, municipal services, nutrition, occupation, socioeconomic and co-morbidities.

Following the data extraction and to help illustrate how the information collected answered the aims and objectives, the results were presented both; (1) numerically, with outbreaks broken down and quantified by disaster, geographic region and pathogen, along with the importance of risk factors and (2) narratively, by synthesising the methods used, the importance of global change and the links between risk factors and possible cascades. The main focus of the results were the risk factors, as the drivers of disease outbreaks, not the region, disaster or disease. We used Pearson’s chi-squared tests (significance level set at *P* ≤ 0.05) to test the significance and residual correlations of disasters, regions, disasters and risk factors and binomial confidence intervals (95% level of confidence) to account for potential limitations due to sample size. We also used hierarchical cluster analysis to show the similarity between risk factor cluster reporting. A distance matrix was first computed, and then hierarchical clustering used to analyse the set of dissimilarities; this was plotted on a dendrogram^67^. All statistical analysis was completed, and figures generated using R version 3.6.2^73^.

## Supplementary Information

Supplementary Information
