# Supplementary material for: Traits and risk factors of post-disaster infectious disease outbreaks: a systematic review
Source: Sci Rep. 2021 Mar 10;11:5616. doi: 10.1038/s41598-021-85146-0 (PMC7970931; doi:10.1038/s41598-021-85146-0)
Supplement: Supplementary file 1 — Supplementary Information [file 41598_2021_85146_MOESM1_ESM.pdf]

Supplementary Information

Traits and risk factors of post-disaster infectious disease outbreaks: a systematic review

Gina E C Charnley, Ilan Kelman, Katy A M Gaythorpe, Kris A Murray

Supplementary Figures

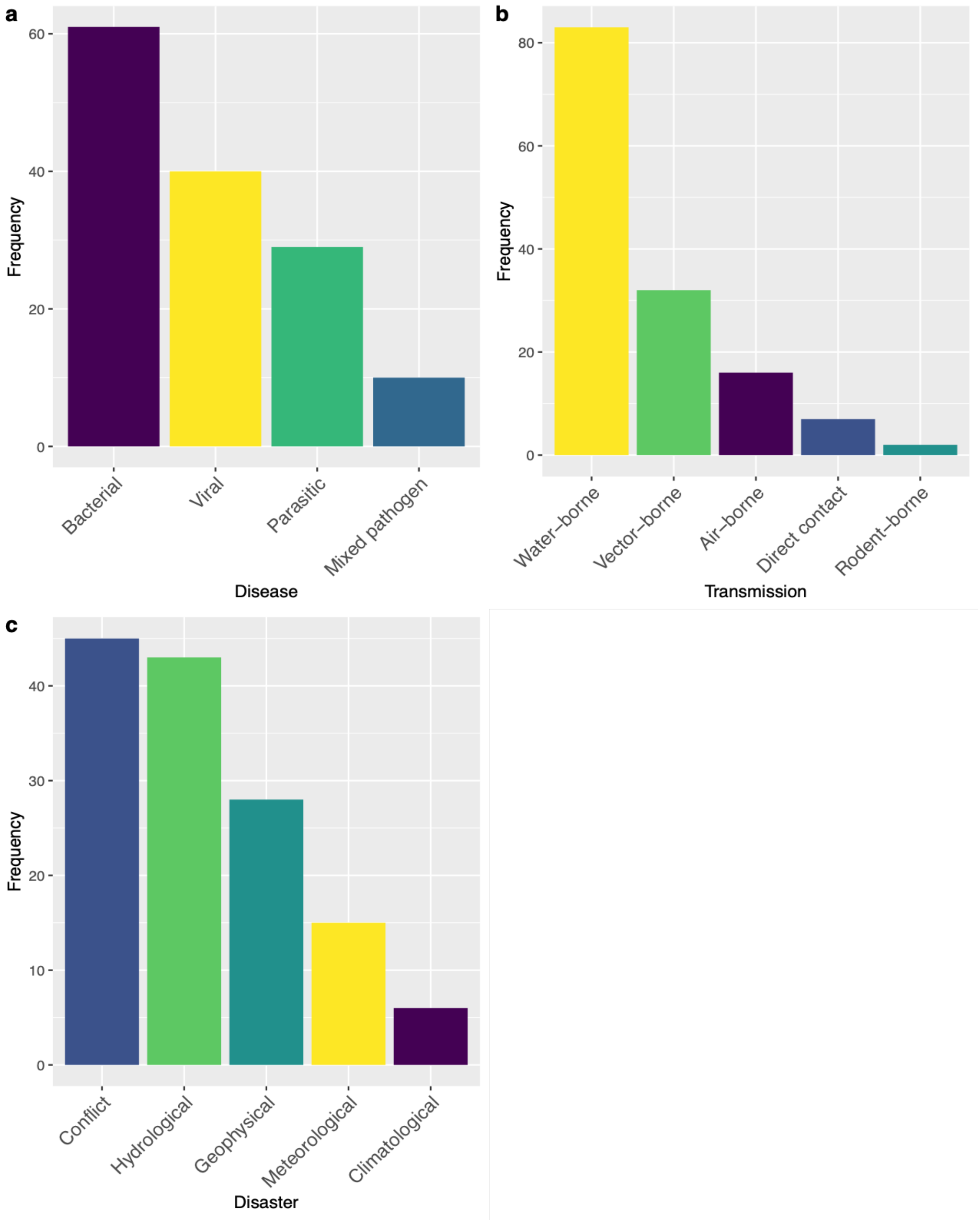

Supplementary Figure 1: Frequencies of reported disaster-related outbreaks separated into a, disease, b, disease transmission (n=140) and c, disaster (n=137)

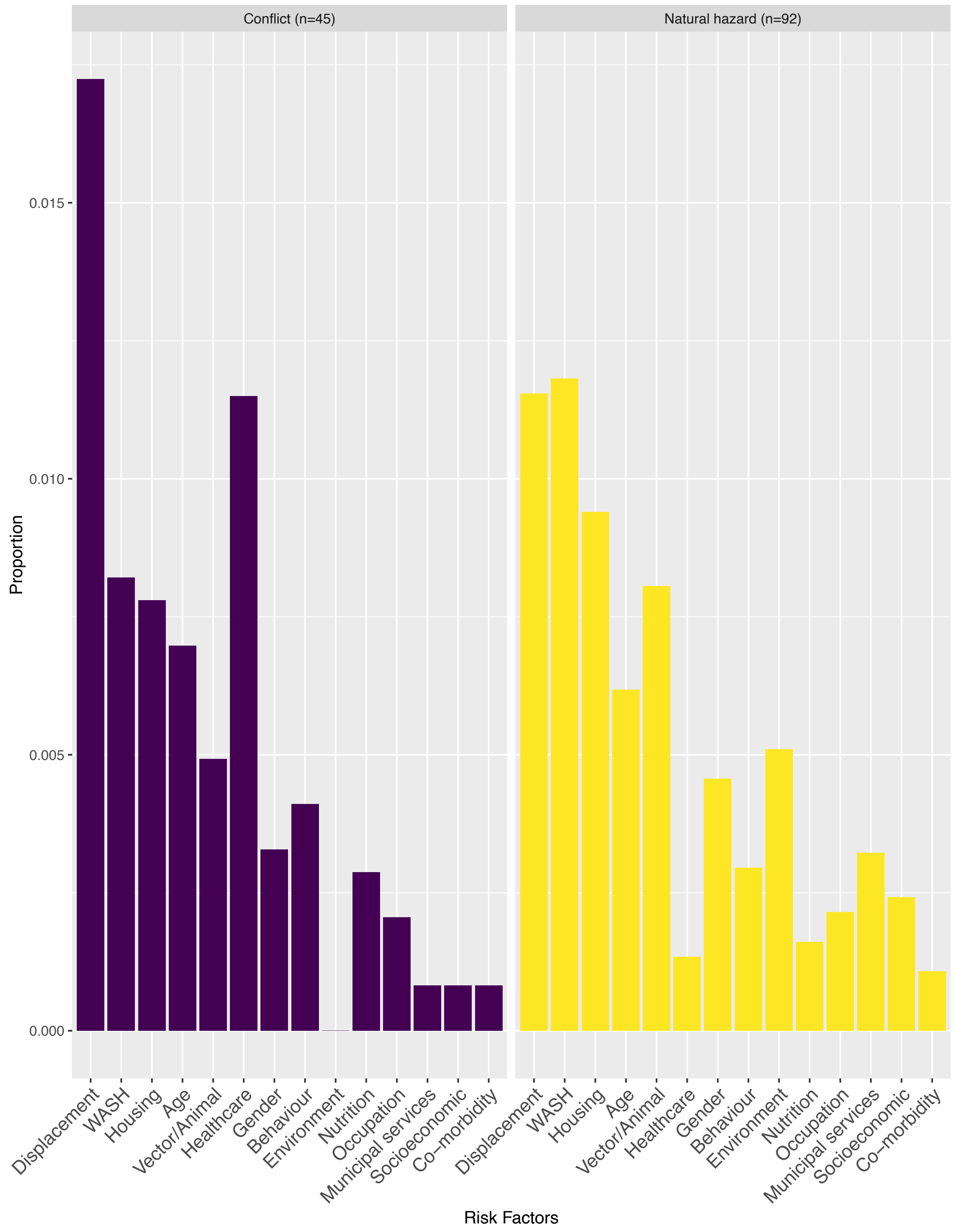

Supplementary Figure 2: Proportions of reported risk factor clusters against conflict and natural hazards

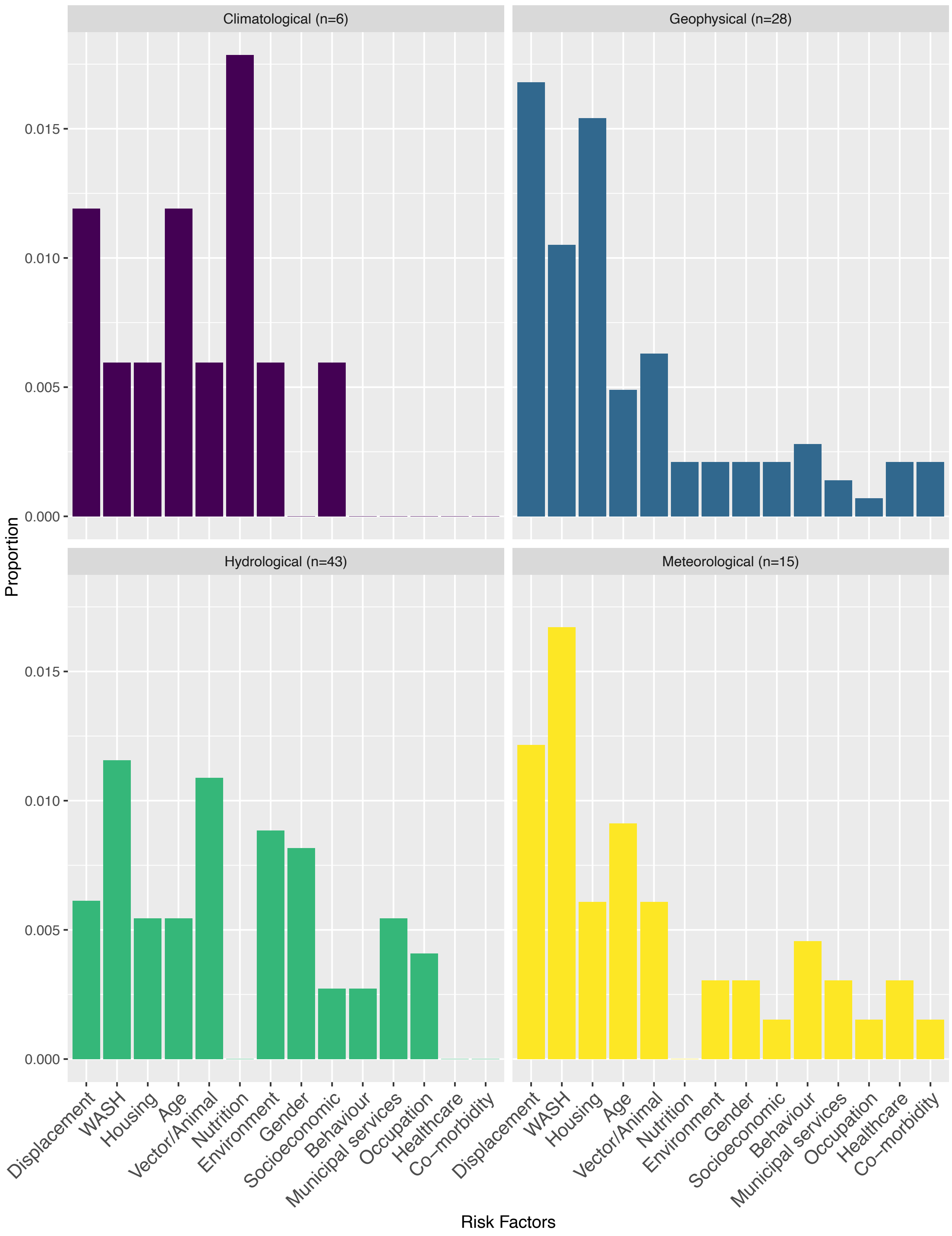

Supplementary Figure 3: Proportions of reported risk factor clusters against the four natural hazards categories

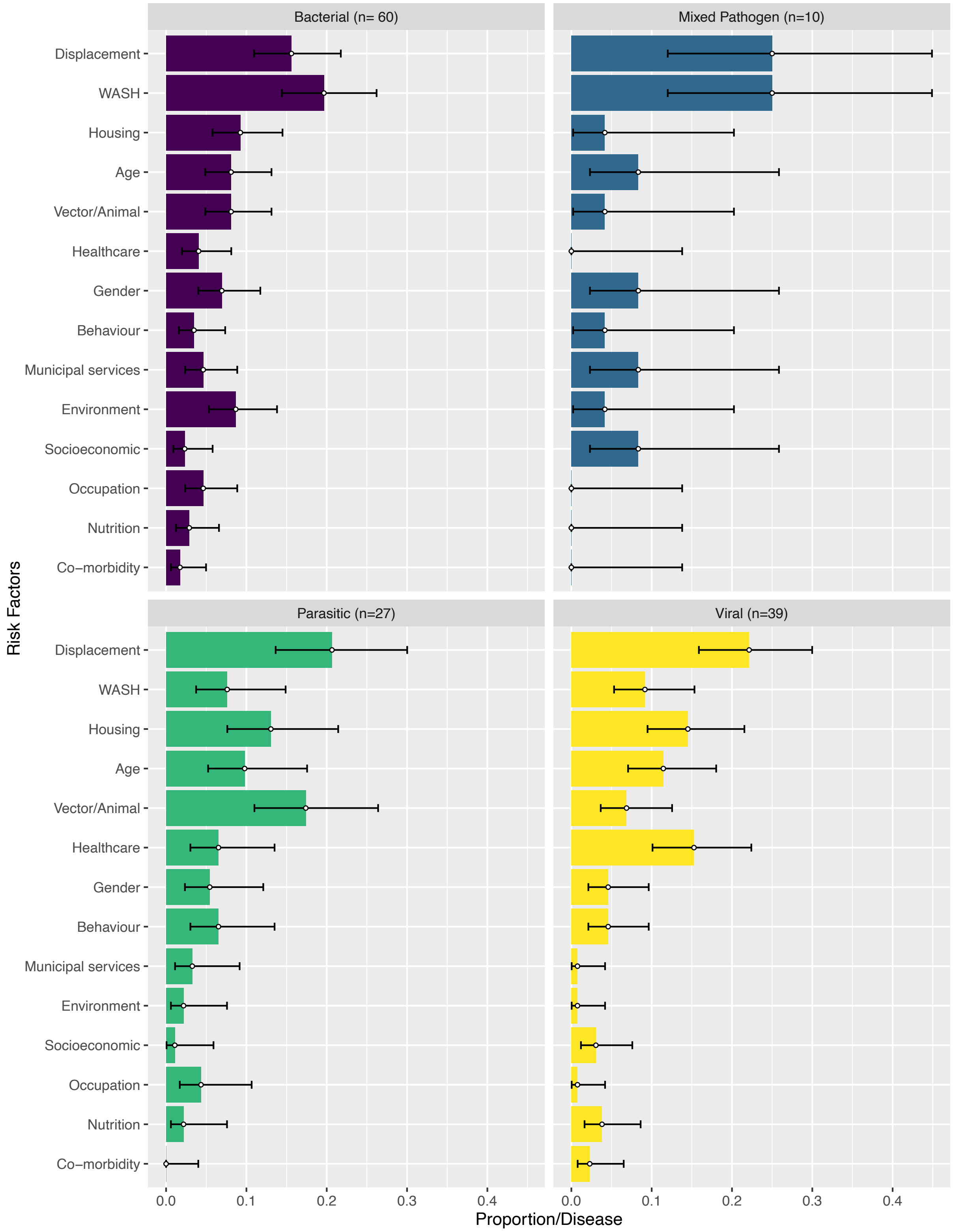

Supplementary Figure 4: Proportions of reported risk factor clusters against disease type with 95% binomial confidence intervals

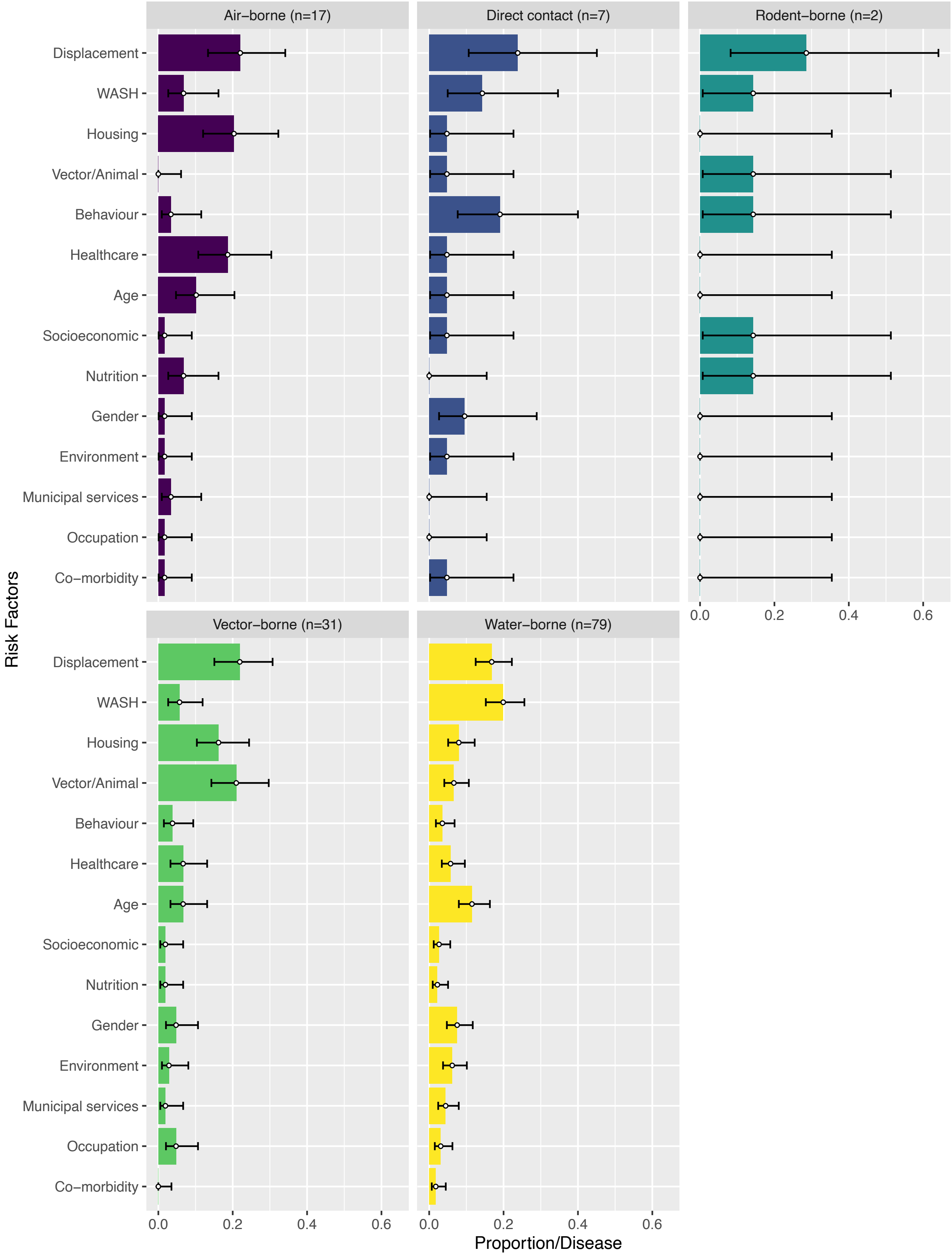

Supplementary Figure 5: Proportions of reported risk factor clusters against disease transmission with 95% binomial confidence intervals

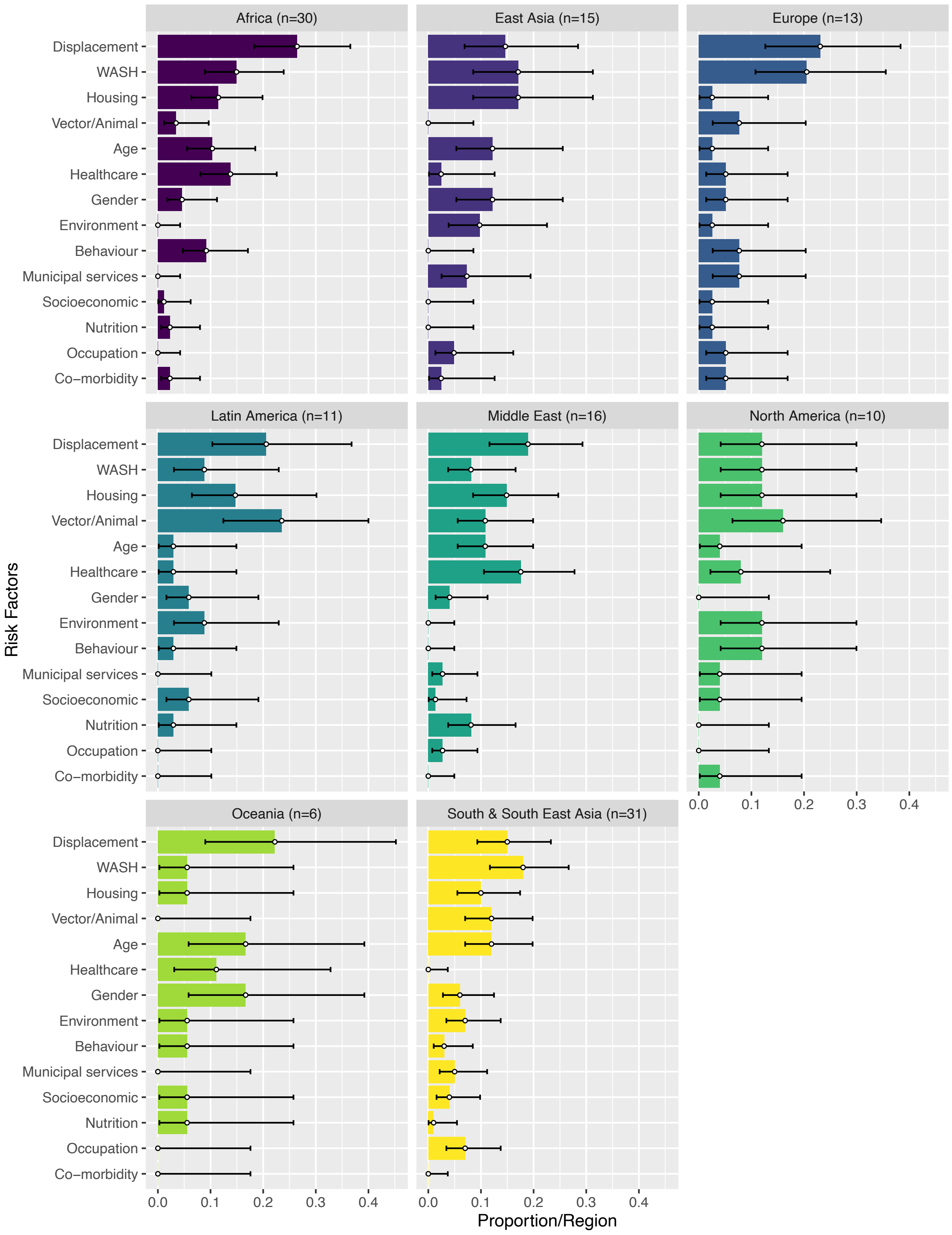

Supplementary Figure 6: Proportions of reported risk factor clusters against the eight region categories with 95% binomial confidence intervals

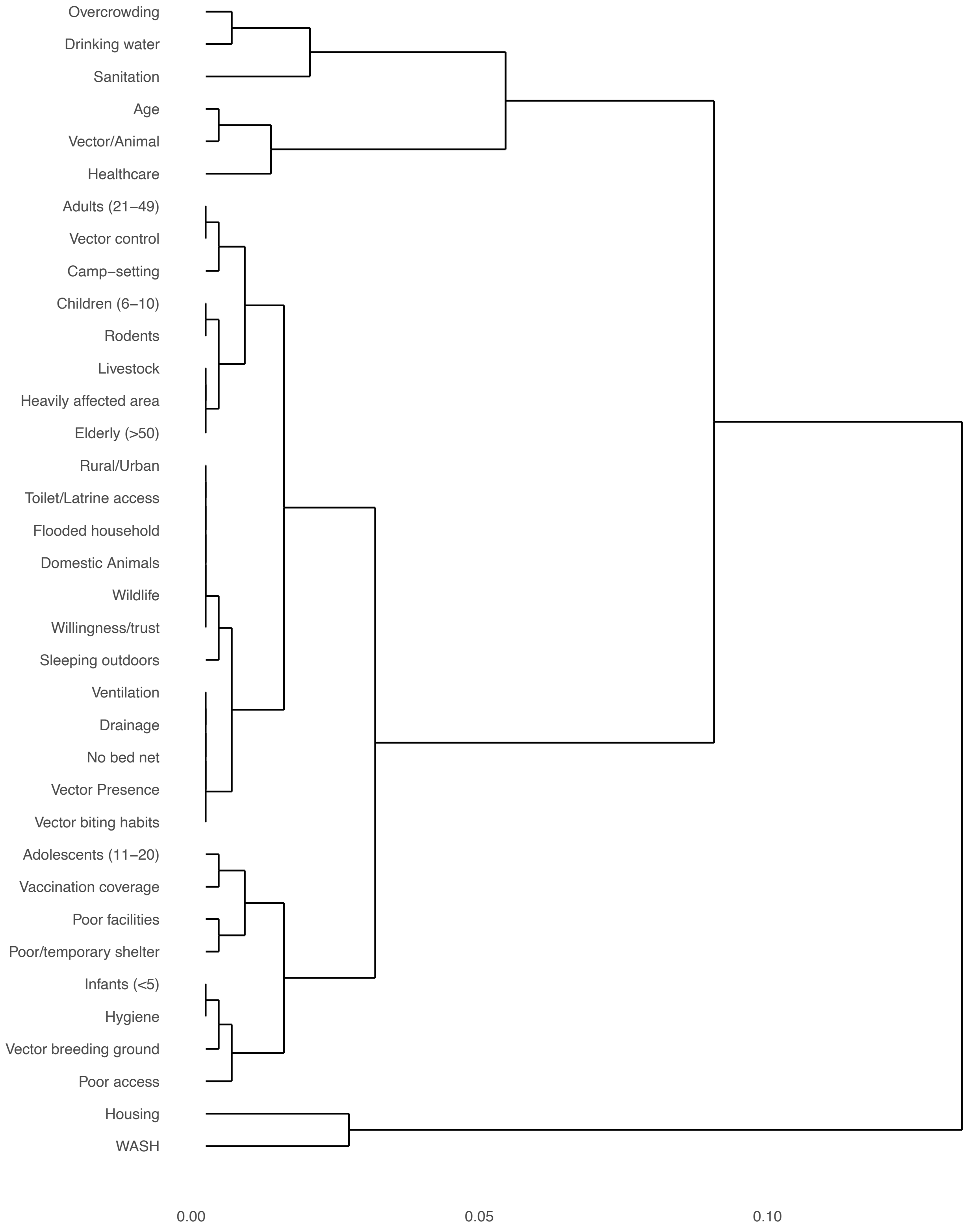

Supplementary Figure 7: Hierarchical cluster analysis of the top five risk factor clusters, broken down into individual risks reported within the cluster.

PRISMA-P (Preferred Reporting Items for Systematic review and Meta-Analysis Protocols) 2015 checklist: recommended items to address in a systematic review protocol*

Section and topic	Item No	Checklist item
**ADMINISTRATIVE INFORMATION**		
Title:		
Identification	1a	Identify the report as a protocol of a systematic review
Update	1b	If the protocol is for an update of a previous systematic review, identify as such
Registration	2	If registered, provide the name of the registry (such as PROSPERO) and registration number
Authors:		
Contact	3a	Provide name, institutional affiliation, e-mail address of all protocol authors; provide physical mailing address of corresponding author
Contributions	3b	Describe contributions of protocol authors and identify the guarantor of the review
Amendments	4	If the protocol represents an amendment of a previously completed or published protocol, identify as such and list changes; otherwise, state plan for documenting important protocol amendments
Support:		
Sources	5a	Indicate sources of financial or other support for the review
Sponsor	5b	Provide name for the review funder and/or sponsor
Role of sponsor or funder	5c	Describe roles of funder(s), sponsor(s), and/or institution(s), if any, in developing the protocol
**INTRODUCTION**		
Rationale	6	Describe the rationale for the review in the context of what is already known
Objectives	7	Provide an explicit statement of the question(s) the review will address with reference to participants, interventions, comparators, and outcomes (PICO)
**METHODS**		
Eligibility criteria	8	Specify the study characteristics (such as PICO, study design, setting, time frame) and report characteristics (such as years considered, language, publication status) to be used as criteria for eligibility for the review
Information sources	9	Describe all intended information sources (such as electronic databases, contact with study authors, trial registers or other grey literature sources) with planned dates of coverage
Search strategy	10	Present draft of search strategy to be used for at least one electronic database, including planned limits, such that it could be repeated
Study records:		
Data management	11a	Describe the mechanism(s) that will be used to manage records and data throughout the review
Selection process	11b	State the process that will be used for selecting studies (such as two independent reviewers) through each phase of the review (that is, screening, eligibility and inclusion in meta-analysis)
Data collection process	11c	Describe planned method of extracting data from reports (such as piloting forms, done independently, in duplicate), any processes for obtaining and confirming data from investigators
Data items	12	List and define all variables for which data will be sought (such as PICO items, funding sources), any pre-planned data assumptions and simplifications
Outcomes and prioritization	13	List and define all outcomes for which data will be sought, including prioritization of main and additional outcomes, with rationale
Risk of bias in individual studies	14	Describe anticipated methods for assessing risk of bias of individual studies, including whether this will be done at the outcome or study level, or both; state how this information will be used in data synthesis
Data synthesis	15a	Describe criteria under which study data will be quantitatively synthesised
	15b	If data are appropriate for quantitative synthesis, describe planned summary measures, methods of handling data and methods of combining data from studies, including any planned exploration of consistency (such as I^2, Kendall's τ)
	15c	Describe any proposed additional analyses (such as sensitivity or subgroup analyses, meta-regression)
	15d	If quantitative synthesis is not appropriate, describe the type of summary planned
Meta-bias(es)	16	Specify any planned assessment of meta-bias(es) (such as publication bias across studies, selective reporting within studies)
Confidence in cumulative evidence	17	Describe how the strength of the body of evidence will be assessed (such as GRADE)

*** It is strongly recommended that this checklist be read in conjunction with the PRISMA-P Explanation and Elaboration (cite when available) for important clarification on the items. Amendments to a review protocol should be tracked and dated.** **The copyright for PRISMA-P (including checklist) is held by the PRISMA-P Group and is distributed under a Creative Commons Attribution Licence 4.0.**

Supplementary Figure 8: PRISMA-P (Preferred Reporting Items for Systematic review and Meta-Analysis Protocols) 2015 checklist: recommended items to address in a systematic review

Supplementary Tables

[1] Bhardwaj, P., Kosambiya, J.K. and DESAI, V.K., 2008. A case control study to explore the risk factors for acquisition of leptospirosis in Surat city, after flood. *Indian journal of medical sciences*, *62*(11).
[2] Hussein, N.R., Balatay, A.A., Saleem, Z.S., Hassan, S.M., Assafi, M.S., Sheikhan, R.S., Amedi, F.R., Hafzullah, S.S., Hafzullah, M.S., Xedr, A.M. and Zebary, M.T., 2019. A clinical study of cutaneous leishmaniasis in a new focus in the Kurdistan region, Iraq. *PloS one*, *14*(5).
[3] Ventura, R.J., Muhi, E., de Los Reyes, V.C., Sucaldito, M.N. and Tayag, E., 2015. A community-based gastroenteritis outbreak after Typhoon Haiyan, Leyte, Philippines, 2013. *Western Pacific surveillance and response journal: WPSAR*, *6*(1), p.1.
[4] Togami, E., Kama, M., Goarant, C., Craig, S.B., Lau, C., Ritter, J.M., Imrie, A., Ko, A.I. and Nilles, E.J., 2018. A large leptospirosis outbreak following successive severe floods in Fiji, 2012. *The American journal of tropical medicine and hygiene*, *99*(4), pp.849-851.
[5] Roy, S., Bhattacharya, D., Ghoshal, S.R., Thanasekaran, K., Bharadwaj, A.P., Singhania, M. and Sugunan, A.P., 2009. Acute diarrhea in children after 2004 tsunami, Andaman Islands. *Emerging Infectious Diseases*, *15*(5), p.849.
[6] McCarthy, M.C., He, J., Hyams, K.C., El-Tigani, A. and Khalid, I.O., 1994. *Acute hepatitis E infection during the 1988 floods in Khartoum, Sudan* (No. NMRI-94-15). NAVAL MEDICAL RESEARCH INST BETHESDA MD.
[7] Yani, F.F., Elfitrimelly, E. and Basir, D., 2010. Acute respiratory infection in children after earthquake on September 30th, 2009 and its related factors in Malalak and Maninjau villages at Agam District West Sumatera. *Paediatric Respiratory Reviews*, (11), p.S111.
[8] Balasubramaniam, S.M. and Roy, G., 2012. An outbreak investigation of measles after the tsunami in Cuddalore district, Tamil Nadu. *Annals of Tropical Medicine and Public Health*, *5*(4), p.291.
[9] Wei, L., XiuLan, M., ZhiHong, L. and WenZheng, G., 2012. An outbreak of bacillary dysentery caused by flash floods polluted water. *Modern Preventive Medicine*, *39*(13), pp.3203-3204. [Abstract only]
[10] Pradutkanchana, S., Pradutkanchana, J., Kanjanapin, W. and Siripaitoon, P., 2002. An outbreak of leptospirosis after severe flood in Hat Yai in 2000. *Journal of Infectious Diseases and Antimicrobial Agents*, *19*(1), pp.9-13.
[11] Hukic, M., Hübschen, J.M., Seremet, M., Salimovic-Besic, I., Mulaomerovic, M., Mehinovic, N., Karakas, S., Charpentier, E. and Muller, C.P., 2012. An outbreak of rubella in the Federation of Bosnia and Herzegovina between December 2009 and May 2010 indicates failure to vaccinate during wartime (1992–1995). *Epidemiology & Infection*, *140*(3), pp.447-453.
[12] Weniger, B.G., Blaser, M.J., Gedrose, J., Lippy, E.C. and Juranek, D.D., 1983. An outbreak of waterborne giardiasis associated with heavy water runoff due to warm weather and volcanic ashfall. *American journal of public health*, *73*(8), pp.868-872.
[13] Zhang, F., Ding, G., Liu, Z., Zhang, C. and Jiang, B., 2016. Association between flood and the morbidity of bacillary dysentery in Zibo City, China: a symmetric bidirectional case-crossover study. *International journal of biometeorology*, *60*(12), pp.1919-1924.
[14] Liu, Z., Lao, J., Zhang, Y., Liu, Y., Zhang, J., Wang, H. and Jiang, B., 2018. Association between floods and typhoid fever in Yongzhou, China: effects and vulnerable groups. *Environmental research*, *167*, pp.718-724.
[15] Fiasca, F., Mattei, A., Vittorini, P., Necozione, S., Appetiti, A., Angelone, A.M. and Fabiani, L., 2018. Bacterial Meningitis Hospitalizations after the 2009 L'Aquila Earthquake: A Retrospective Observational Study. *Asian J Epidemiol*, *11*(1), pp.46-51.
[16] Tanabe, K., Nakamura, S. and Kunii, O., 1999. Bacteriological survey of diarrheal epidemics in the 1998 Bangladesh floods. *Kansenshogaku zasshi. The Journal of the Japanese Association for Infectious Diseases*, *73*(9), pp.918-922. [Abstract only]
[17] Montero, D., Vidal, M., Pardo, M., Torres, A., Kruger, E., Farfán, M., O'Ryan, M., Luo, Q., Fleckenstein, J., Del Canto, F. and Vidal, R., 2017. Characterization of enterotoxigenic Escherichia coli strains isolated from the massive multi-pathogen gastroenteritis outbreak in the Antofagasta region following the Chilean earthquake, 2010. *Infection, Genetics and Evolution*, *52*, pp.26-29.
[18] Centers for Disease Control and Prevention (CDC, 2003. Cholera epidemic after increased civil conflict--Monrovia, Liberia, June-September 2003. *MMWR. Morbidity and mortality weekly report*, *52*(45), p.1093.
[19] Fredrick, T., Ponnaiah, M., Murhekar, M.V., Jayaraman, Y., David, J.K., Vadivoo, S. and Joshua, V., 2015. Cholera outbreak linked with lack of safe water supply following a tropical cyclone in Pondicherry, India, 2012. *Journal of health, population, and nutrition*, *33*(1), p.31.

[20] Yuan, X., 2018. Concern over reported number of measles cases in Yemen. *The Lancet*, *391*(10133), p.1886.
[21] Rosewell, A., Clark, G., Mabong, P., Ropa, B., Posanai, E., Man, N.W., Dutta, S.R., Wickramasinghe, W., Qi, L., Ng, J.C. and Mola, G., 2013. Concurrent outbreaks of cholera and peripheral neuropathy associated with high mortality among persons internally displaced by a volcanic eruption. *PloS one*, *8*(9).
[22] Norris, A., Hachey, K., Curtis, A. and Bourdeaux, M., 2016. Crippling violence: Conflict and incident polio in Afghanistan. *PLoS one*, *11*(3).
[23] Al-Salem, W.S., Pigott, D.M., Subramaniam, K., Haines, L.R., Kelly-Hope, L., Molyneux, D.H., Hay, S.I. and Acosta-Serrano, A., 2016. Cutaneous leishmaniasis and conflict in Syria. *Emerging infectious diseases*, *22*(5), p.931.
[24] Fanany, D., 2012. Dengue hemorrhagic fever and natural disaster: the case of Padang, West Sumatra. *International Journal of Collaborative Research on Internal Medicine & Public Health*, *4*(5), pp.0-0.
[25] Alghazali, K.A., Teoh, B.T., Loong, S.K., Sam, S.S., Che-Mat-Seri, N.A.A., Samsudin, N.I., Yaacob, C.N., Azizan, N.S., Oo, A., Baharudin, N.A. and Tan, K.K., 2019. Dengue outbreak during ongoing civil war, Taiz, Yemen. *Emerging infectious diseases*, *25*(7), p.1397.
[26] Schwartz, B.S., Harris, J.B., Khan, A.I., Larocque, R.C., Sack, D.A., Malek, M.A., Faruque, A.S., Qadri, F., Calderwood, S.B., Luby, S.P. and Ryan, E.T., 2006. Diarrheal epidemics in Dhaka, Bangladesh, during three consecutive floods: 1988, 1998, and 2004. *The American journal of tropical medicine and hygiene*, *74*(6), pp.1067-1073.
[27] Dureab, F., Al-Sakkaf, M., Ismail, O., Kuunibe, N., Krisam, J., Müller, O. and Jahn, A., 2019. Diphtheria outbreak in Yemen: the impact of conflict on a fragile health system. *Conflict and health*, *13*(1), p.19.
[28] Chretien, J.P., Anyamba, A., Bedno, S.A., Breiman, R.F., Sang, R., Sergon, K., Powers, A.M., Onyango, C.O., Small, J., Tucker, C.J. and Linthicum, K.J., 2007. Drought-associated chikungunya emergence along coastal East Africa. *The American journal of tropical medicine and hygiene*, *76*(3), pp.405-407.
[29] McPake, B., Witter, S., Ssali, S., Wurie, H., Namakula, J. and Ssengooba, F., 2015. Ebola in the context of conflict affected states and health systems: case studies of Northern Uganda and Sierra Leone. *Conflict and health*, *9*(1), p.23.
[30] Claude, K.M., Underschultz, J. and Hawkes, M.T., 2018. Ebola virus epidemic in war-torn eastern DR Congo. *The Lancet*, *392*(10156), pp.1399-1401.
[31] Tauxe, R.V., Holmberg, S.D., Dodin, A., Wells, J.V. and Blake, P.A., 1988. Epidemic cholera in Mali: high mortality and multiple routes of transmission in a famine area. *Epidemiology & Infection*, *100*(2), pp.279-289.
[32] Karim, A.M., Hussain, I., Malik, S.K., Lee, J.H., Cho, I.H., Kim, Y.B. and Lee, S.H., 2016. Epidemiology and clinical burden of malaria in the war-torn area, Orakzai Agency in Pakistan. *PLoS neglected tropical diseases*, *10*(1).
[33] Hashizume, M., Wagatsuma, Y., Faruque, A.S., Hayashi, T., Hunter, P.R., Armstrong, B. and Sack, D.A., 2008. Factors determining vulnerability to diarrhoea during and after severe floods in Bangladesh. *Journal of Water and Health*, *6*(3), pp.323-332.
[34] Sidley, P., 2008. Floods in Mozambique result in cholera and displacement. *BMJ: British Medical Journal*, *336*(7642), pp.471-471.
[35] Lora-Suarez, F., Marin-Vasquez, C., Loango, N., Gallego, M., Torres, E., Gonzalez, M.M., Castaño-Osorio, J.C. and Gómez-Marín, J.E., 2002. Giardiasis in children living in post-earthquake camps from Armenia (Colombia). *BMC Public Health*, *2*(1), p.5.
[36] Bugert, J.J., Welzel, T.M., Zeier, M. and Darai, G., 1999. Hantavirus infection—haemorrhagic fever in the Balkans—potential nephrological hazards in the Kosovo war. *Nephrology Dialysis Transplantation*, *14*(8), pp.1843-1844.
[37] Kaddoura, M., Allaham, R., Abubakar, A., Ezzeddine, A., Barakat, A., Mala, P. and Zaraket, H., 2020. Hepatitis A Virus Genotype IB Outbreak among Internally Displaced Persons, Syria. *Emerging infectious diseases*, *26*(2), p.369.
[38] Wei, W.A.N.G., Xun, H.M., Zhou, M.G., Jiang, B.F., Wang, S.W., Qing, G.U.O., Kang, R.H., Xin, W.A.N.G., Gifty, M. and Wei, M.A., 2015. Impacts of typhoon 'Koppu'on infectious diarrhea in Guangdong Province, China. *Biomedical and Environmental Sciences*, *28*(12), pp.920-923.
[39] Caillouët, K.A., Michaels, S.R., Xiong, X., Foppa, I. and Wesson, D.M., 2008. Increase in West Nile neuroinvasive disease after hurricane Katrina. *Emerging infectious diseases*, *14*(5), p.804.

[40] Schousboe, M., Lynds, J. and Ambrose, C., 2013. Increased incidence of Escherichia coli bacteremia post-Christchurch earthquake 2011: possible associations. *Prehospital and disaster medicine*, *28*(3), pp.202-209.

[41] Jones, F.K., Ko, A.I., Becha, C., Joshua, C., Musto, J., Thomas, S., Ronsse, A., Kirkwood, C.D., Sio, A., Aumua, A. and Nilles, E.J., 2016. Increased rotavirus prevalence in diarrheal outbreak precipitated by localized flooding, Solomon Islands, 2014. *Emerging infectious diseases*, *22*(5), p.875.

[42] Chen, W., Yang, X., Huang, X., Zhang, Y., Cai, S., Liu, J., Fu, Y. and Li, S., 2000. Influence of flood in 1998 on schistosomiasis epidemic. *Chinese Journal of Schistosomiasis Control*, *12*(4), pp.202-205. [Abstract only]

[43] Kalthan, E., Tenguere, J., Ndjapou, S.G., Koyazengbe, T.A., Mbomba, J., Marada, R.M., Rombebe, P., Yangueme, P., Babamingui, M., Sambella, A. and Nakoune, E.R., 2018. Investigation of an outbreak of monkeypox in an area occupied by armed groups, Central African Republic. *Medecine et maladies infectieuses*, *48*(4), pp.263-268.

[44] Enbiale, W. and Ayalew, A., 2018. Investigation of a scabies outbreak in drought-affected areas in Ethiopia. *Tropical medicine and infectious disease*, *3*(4), p.114.

[45] Shaffer, J.G., Grant, D.S., Schieffelin, J.S., Boisen, M.L., Goba, A., Hartnett, J.N., Levy, D.C., Yenni, R.E., Moses, L.M., Fullah, M. and Momoh, M., 2014. Lassa fever in post-conflict Sierra Leone. *PLoS Negl Trop Dis*, *8*(3), p.e2748.

[46] Zúñiga Carrasco, I.R., Beatriz, B., Ángel, B., Williams, M. and Miguel, D., 2011. Later leptospirosis after flood in Tabasco, Mexico, 2007. *Enfermedades Infecciosas y Microbiología*, *31*(1), pp.33-37. [Abstract only]

[47] Chiu, C.H., Wang, Y.C., Yang, Y.S. and Chang, F.Y., 2009. Leptospirosis after typhoon in Taiwan. *J Med Sci*, *29*(3), pp.131-134.

[48] Pellizzer, P., Todescato, A., Benedetti, P., Colussi, P., Conz, P. and Cinco, M., 2006. Leptospirosis following a flood in the Veneto area, North-east Italy. *Annali di igiene: medicina preventiva e di comunita*, *18*(5), pp.453-456. [Abstract only]

[49] Smith, J.K., Young, M.M., Wilson, K.L. and Craig, S.B., 2013. Leptospirosis following a major flood in Central Queensland, Australia. *Epidemiology & Infection*, *141*(3), pp.585-590.

[50] Supe, A., Khetarpal, M., Naik, S. and Keskar, P., 2018. Leptospirosis following heavy rains in 2017 in Mumbai: Report of large-scale community chemoprophylaxis. *The National medical journal of India*, *31*(1), p.19.

[51] Easton, A., 1999. Leptospirosis in Philippine floods.

[52] Gaynor, K., Katz, A.R., Park, S.Y., Nakata, M., Clark, T.A. and Effler, P.V., 2007. Leptospirosis on Oahu: an outbreak associated with flooding of a university campus. *The American journal of tropical medicine and hygiene*, *76*(5), pp.882-886.

[53] Radi, M.F.M., Hashim, J.H., Jaafar, M.H., Hod, R., Ahmad, N., Nawi, A.M., Baloch, G.M., Ismail, R. and Ayub, N.I.F., 2018. Leptospirosis outbreak after the 2014 major flooding event in Kelantan, Malaysia: a spatial-temporal analysis. *The American journal of tropical medicine and hygiene*, *98*(5), pp.1281-1295.

[54] World Health Organization, 2000. Leptospirosis, India: report of the investigation of a post-cyclone outbreak in Orissa, November 1999. *Weekly Epidemiological Record= Relevé épidémiologique hebdomadaire*, *75*(27), pp.217-223.

[55] Salahi-Moghaddam, A., Habibi-Nokhandam, M. and Fuentes, M.V., 2011. Low-altitude outbreaks of human fascioliasis related with summer rainfall in Gilan province, Iran. *Geospatial health*, pp.133-136.

[56] Wiwanitkit, V., 2007. Correspondence-Malaria and dengue infection after Tsunami in Southern Thailand. *Tropical doctor*, *37*(3), pp.194-194.

[57] Manimunda, S.P., Shah, W.A., Shriram, A.N., Sugunan, A.P., Titus, E., Charles, A., Sur, S.K. and Vijayachari, P., 2009. Malaria in Car Nicobar Island in the aftermath of the tsunami: Some observations. *Natl Med J India*, *22*(4), pp.217-218.

[58] Snuparek, J., 1947. Malaria in Czechoslovakia after World War II. *The Journal of Parasitology*, *33*(6), pp.506-508.

[59] GARFIELD, R.M., PRADO, E., GATES, J.R. and VERMUND, S.H., 1989. Malaria in Nicaragua: community-based control efforts and the impact of war. *International journal of epidemiology*, *18*(2), pp.434-439.

[60] Baomar, A.T. and Mohamed, A.G., 2000. Malaria outbreak in a malaria-free region in Oman 1998: unknown impact of civil war in Africa. *Public Health*, *114*(6), pp.480-483.

[61] Muriuki, D., Hahn, S., Hexom, B. and Allan, R., 2012. Cross-sectional survey of malaria prevalence in tsunami-affected districts of Aceh Province, Indonesia. *International journal of emergency medicine*, *5*(1), p.11.

[62] Valente, F., Otten, M., Balbina, F., Van de Weerdt, R., Chezzi, C., Eriki, P., Van-Dúnnen, J. and Okwo Bele, J.M., 2000. Massive outbreak of poliomyelitis caused by type-3 wild poliovirus in Angola in 1999. *Bulletin of the World Health Organization*, *78*, pp.339-346.

[63] Tekpa, G., H. S. C., Diemer, J. D. D., Longo, C. M. Pamatika., Z. Dalengat-Vogbia. and B. Koffi., 2019. Measles Epidemic in Post-Conflict Period in Rural Central African Republic. *Médecine d'Afrique Noire, 66*(7), pp.387–92. [Abstract only]

[64] Ahmad, K., 2000. Measles epidemic sweeps through Afghanistan. *The Lancet*, *355*(9213), p.1439.

[65] Cambaza, E., Mongo, E., Anapakala, E., Nhambire, R., Singo, J. and Machava, E., 2019. Outbreak of cholera due to Cyclone Kenneth in northern Mozambique, 2019. *International journal of environmental research and public health*, *16*(16), p.2925.

[66] Centers for Disease Control (CDC, 1983. Outbreak of diarrheal illness associated with a natural disaster--Utah. *MMWR. Morbidity and mortality weekly report*, *32*(50), p.662.

[67] Gertler, M., Dürr, M., Renner, P., Poppert, S., Askar, M., Breidenbach, J., Frank, C., Preußel, K., Schielke, A., Werber, D. and Chalmers, R., 2015. Outbreak of Cryptosporidium hominis following river flooding in the city of Halle (Saale), Germany, August 2013. *BMC infectious diseases*, *15*(1), p.88.

[68] Vanasco, N.B., Fusco, S., Zanuttini, J.C., Manattini, S., Dalla, M.F., Prez, J., Cerrano, D. and Sequeira, M.D., 2002. Outbreak of human leptospirosis after a flood in Reconquista, Santa Fe, 1998. *Revista Argentina de microbiologia*, *34*(3), pp.124-131. [Abstract only]

[69] Al-shere, T.A., Ujiie, M., Suzuki, M., Salva, E., Belo, M.C.P., Koizumi, N., Yoshimatsu, K., Schmidt, W.P., Marte, S., Dimaano, E.M. and Villarama, J.B., 2012. Outbreak of leptospirosis after flood, the Philippines, 2009. *Emerging infectious diseases*, *18*(1), p.91.

[70] Nomura, K., Murai, H., Nakahashi, T., Mashiba, S., Watoh, Y., Takahashi, T. and Morimoto, S., 2008. Outbreak of norovirus gastroenteritis in elderly evacuees after the 2007 Noto Peninsula earthquake in Japan. *Journal of the American Geriatrics Society*, *56*(2), pp.361-363.

[71] Takahashi, H., Fujimura, S., Ubukata, S., Sato, E., Shoji, M., Utagawa, M., Kikuchi, T. and Watanabe, A., 2012. Pneumonia after earthquake, Japan, 2011. *Emerging infectious diseases*, *18*(11), p.1909.

[72] Kamadjeu, R., Mahamud, A., Webeck, J., Baranyikwa, M.T., Chatterjee, A., Bile, Y.N., Birungi, J., Mbaeyi, C. and Mulugeta, A., 2014. Polio outbreak investigation and response in Somalia, 2013. *The Journal of infectious diseases*, *210*(suppl_1), pp.S181-S186.

[73] Sáenz, R., Bissell, R.A. and Paniagua, F., 1995. Post-disaster malaria in Costa Rica. *Prehospital and disaster medicine*, *10*(3), pp.154-160.

[74] Fakoorziba, M.R., Baseri, A., Eghbal, F., Rezaee, S., Azizi, K. and Moemenbellah-Fard, M.D., 2011. Post-earthquake outbreak of cutaneous leishmaniasis in a rural region of southern Iran. *Annals of Tropical Medicine & Parasitology*, *105*(3), pp.217-224.

[75] Karmakar, S., Rathore, A.S., Kadri, S.M., Dutt, S., Khare, S. and Lal, S., 2008. Post-earthquake outbreak of rotavirus gastroenteritis in Kashmir (India): An epidemiological analysis. *Public health*, *122*(10), pp.981-989.

[76] Ortiz, M.R., Le, N.K., Sharma, V., Hoare, I., Quizhpe, E., Teran, E., Naik, E., Salihu, H.M. and Izurieta, R., 2017. Post-earthquake Zika virus surge: Disaster and public health threat amid climatic conduciveness. *Scientific reports*, *7*(1), pp.1-10.

[77] Hatta, M., Endo, S., Tokuda, K., Kunishima, H., Arai, K., Yano, H., Ishibashi, N., Aoyagi, T., Yamada, M., Inomata, S. and Kanamori, H., 2012. Post-tsunami outbreaks of influenza in evacuation centers in Miyagi Prefecture, Japan. *Clinical Infectious Diseases*, *54*(1), pp.e5-e7.

[78] Frawley., A. A., Schafer, I. J., Galloway, R., Artus, A. and Ratard, R. C., 2017. Postflooding leptospirosis - Louisiana, 2016. MMWR. Morbidity and mortality weekly report, 66(42), pp.1158-1159.

[79] Xu, X., Ding, G., Zhang, Y., Liu, Z., Liu, Q. and Jiang, B., 2017. Quantifying the impact of floods on bacillary dysentery in Dalian city, China, from 2004 to 2010. *Disaster medicine and public health preparedness*, *11*(2), pp.190-195.

[80] Liu, X., Liu, Z., Zhang, Y. and Jiang, B., 2016. Quantitative analysis of burden of bacillary dysentery associated with floods in Hunan, China. *Science of the Total Environment*, *547*, pp.190-196.

[81] Agampodi, S.B., Dahanayaka, N.J., Bandaranayaka, A.K., Perera, M., Priyankara, S., Weerawansa, P., Matthias, M.A. and Vinetz, J.M., 2014. Regional differences of leptospirosis in Sri Lanka: observations from a flood-associated outbreak in 2011. *PLoS neglected tropical diseases*, *8*(1).

[82] Mbaeyi, C., Ryan, M.J., Smith, P., Mahamud, A., Farag, N., Haithami, S., Sharaf, M., Jorba, J.C. and Ehrhardt, D., 2017. Response to a large polio outbreak in a setting of conflict—Middle East, 2013–2015. *MMWR. Morbidity and mortality weekly report*, *66*(8), p.227.

[83] Moore, A., Richer, M., Enrile, M., Losio, E., Roberts, J. and Levy, D., 1999. Resurgence of sleeping sickness in Tambura County, Sudan. *The American journal of tropical medicine and hygiene*, *61*(2), pp.315-318.

[84] Sur, D., Dutta, P., Nair, G.B. and Bhattacharya, S.K., 2000. Severe cholera outbreak following floods in a northern disrict of West Bengal. *Indian Journal of Medical Research*, *112*, p.178. [Abstract only]

[85] Mamova, A., Balazova, M., Sladeckova, V., Dobrodenkova, S., Bartkoviak, M., Benca, J., Michalikova, L., Kulkova, N., Brnova, J., Mikolasova, G. and Kafkova, J., 2015. Severe malaria including cerebral malaria among 3707 admissions in South Sudanese hospital for internally displaced population after tribual conflicts in 2012-2013 (note). *CLINICAL SOCIAL WORK*, p.65.

[86] Suzuki, M., Uwano, C., Ohrui, T., Ebihara, T., Yamasaki, M., Asamura, T., Tomita, N., Kosaka, Y., Furukawa, K. and Arai, H., 2011. Shelter‐acquired pneumonia after a catastrophic earthquake in Japan. *Journal of the American Geriatrics Society*, *59*(10), pp.1968-1970.

[87] Naranjo, M., Suárez, M., Fernández, C., Amador, N., González, M., Batista, N., González, I., Valdés, Y., Infante, J.F. and Sierra, G., 2008. Study of a leptospirosis outbreak in Honduras following Hurricane Mitch and prophylactic protection of the vax-SPIRAL® vaccine. *MEDICC review*, *10*(3), pp.38-42.

[88] Hassan, O.A., Ahlm, C., Sang, R. and Evander, M., 2011. The 2007 rift valley fever outbreak in Sudan. *PLoS neglected tropical diseases*, *5*(9).

[89] Liu, X., Liu, Z., Zhang, Y. and Jiang, B., 2017. The effects of floods on the incidence of bacillary dysentery in Baise (Guangxi Province, China) from 2004 to 2012. *International journal of environmental research and public health*, *14*(2), p.179.

[90] Vahaboglu, H., Gundes, S., Karadenizli, A., Mutlu, B., Cetin, S., Kolayli, F., Coskunkan, F. and Dündar, V., 2000. Transient increase in diarrheal diseases after the devastating earthquake in Kocaeli, Turkey: results of an infectious disease surveillance study. *Clinical infectious diseases*, *31*(6), pp.1386-1389.

[91] Reintjes, R., Dedushaj, I., Gjini, A., Jorgensen, T.R., Cotter, B., Lieftucht, A., D'Ancona, F., Dennis, D.T., Kosoy, M.A., Mulliqi-Osmani, G. and Grunow, R., 2002. Tularemia outbreak investigation in Kosovo: case control and environmental studies. *Emerging infectious diseases*, *8*(1), p.69.

[92] Bradarić, N., Punda-Polić, V., Ivić, I., Grgić, D. and Radošević, N., 1996. Two outbreaks of typhoid fever related to the war in Bosnia and Herzegovina. *European journal of epidemiology*, *12*(4), pp.409-412.

[93] Su, H.P., Chan, T.C. and Chang, C.C., 2011. Typhoon-related leptospirosis and melioidosis, Taiwan, 2009. *Emerging infectious diseases*, *17*(7), p.1322.

[94] O'Connor, K.A., Cartwright, E., Loharikar, A., Routh, J., Gaines, J., Fouché, M.D.B., Jean-Louis, R., Ayers, T., Johnson, D., Tappero, J.W. and Roels, T.H., 2011. Risk factors early in the 2010 cholera epidemic, Haiti. *Emerging infectious diseases*, *17*(11), p.2136.

[95] Centers for Disease Control and Prevention (CDC, 2005. Vibrio illnesses after Hurricane Katrina--multiple states, August-September 2005. *MMWR. Morbidity and mortality weekly report*, *54*(37), p.928.

[96] Gorski, S., Collin, S.M., Ritmeijer, K., Keus, K., Gatluak, F., Mueller, M. and Davidson, R.N., 2010. Visceral leishmaniasis relapse in Southern Sudan (1999–2007): a retrospective study of risk factors and trends. *PLoS neglected tropical diseases*, *4*(6).

[97] Punda-Polić, V., Kraljević, K.Š. and Bradarić, N., 2007. War-associated cases of typhoid fever imported to Split-Dalmatia County (Croatia). *Military medicine*, *172*(10), pp.1096-1098.

[98] Bhunia, R. and Ghosh, S., 2011. Waterborne cholera outbreak following cyclone Aila in Sundarban area of West Bengal, India, 2009. *Transactions of the Royal Society of Tropical Medicine and Hygiene*, *105*(4), pp.214-219.

[99] Dureab, F.A., Shibib, K., Al-Yousufi, R. and Jahn, A., 2018. Yemen: cholera outbreak and the ongoing armed conflict. *The Journal of Infection in Developing Countries*, *12*(05), pp.397-403.

[100] Qadri, F., Khan, A.I., Faruque, A.S.G., Begum, Y.A., Chowdhury, F., Nair, G.B., Salam, M.A., Sack, D.A. and Svennerholm, A.M., 2005. Enterotoxigenic Escherichia coli and Vibrio cholerae diarrhea, Bangladesh, 2004. *Emerging infectious diseases*, *11*(7), p.1104.

[101] De Man, H., Gras, L.M., Schimmer, B., Friesema, I.H.M., HUSMAN, A.D.R. and Van Pelt, W., 2016. Gastrointestinal, influenza-like illness and dermatological complaints following exposure to floodwater: a cross-sectional survey in The Netherlands. *Epidemiology & Infection*, *144*(7), pp.1445-1454.

[102] Ford, L.B., 2007. Civil conflict and sleeping sickness in Africa in general and Uganda in particular. *Conflict and health*, *1*(1), p.6.

[103] Shikanga, O.T., Mutonga, D., Abade, M., Amwayi, S., Ope, M., Limo, H., Mintz, E.D., Quick, R.E., Breiman, R.F. and Feikin, D.R., 2009. High mortality in a cholera outbreak in western Kenya after post-election violence in 2008. *The American journal of tropical medicine and hygiene*, *81*(6), pp.1085-1090

[104] Marlet, M.V.L., Sang, D.K., Ritmeijer, K., Muga, R.O., Onsongo, J. and Davidson, R.N., 2003. Emergence or re-emergence of visceral leishmaniasis in areas of Somalia, northeastern Kenya, and south-eastern Ethiopia in 2000–2001. *Transactions of the Royal Society of Tropical Medicine and Hygiene*, *97*(5), pp.515-518.

[105] Bastola, A., Marahatta, S.B., Jha, S. and Pant, N., 2017. Aftermath earthquake in Nepal: burden of scrub typhus cases and their presentations. *Journal of Tropical Diseases*, *5*(1), p.236.

[106] Trevejo, R.T., Rigau-Pérez, J.G., Ashford, D.A., McClure, E.M., Jarquín-González, C., Amador, J.J., de los Reyes, J.O., Gonzalez, A., Zaki, S.R., Shieh, W.J. and McLean, R.G., 1998. Epidemic leptospirosis associated with pulmonary hemorrhage—Nicaragua, 1995. *The Journal of infectious diseases*, *178*(5), pp.1457-1463.

[107] Ding, G., Zhang, Y., Gao, L., Ma, W., Li, X., Liu, J., Liu, Q. and Jiang, B., 2013. Quantitative analysis of burden of infectious diarrhea associated with floods in northwest of Anhui Province, China: a mixed method evaluation. *PLOS one*, *8*(6).

[108] Reacher, M., McKenzie, K., Lane, C., Nichols, T., Kedge, I., Iversen, A., Hepple, P., Walter, T., Laxton, C. and Simpson, J., 2004. Health impacts of flooding in Lewes: a comparison of reported gastrointestinal and other illness and mental health in flooded and non-flooded households. *Communicable Disease and Public Health*, *7*, pp.39-46.

[109] Tricou, V., Pagonendji, M., Manengu, C., Mutombo, J., Mabo, R.O. and Gouandjika-Vasilache, I., 2013. Measles outbreak in Northern Central African Republic 3 years after the last national immunization campaign. *BMC infectious diseases*, *13*(1), p.103.

[110] Dechet, A.M., Parsons, M., Rambaran, M., Mohamed-Rambaran, P., Florendo-Cumbermack, A., Persaud, S., Baboolal, S., Ari, M.D., Shadomy, S.V., Zaki, S.R. and Paddock, C.D., 2012. Leptospirosis outbreak following severe flooding: a rapid assessment and mass prophylaxis campaign; Guyana, January–February 2005. *PLoS One*, *7*(7).

[111] Maskey, M., Shastri, J.S., Saraswathi, K., Surpam, R. and Vaidya, N., 2006. Leptospirosis in Mumbai: post-deluge outbreak 2005. *Indian journal of medical microbiology*, *24*(4), p.337.

[112] Raoult, D., Ndihokubwayo, J.B., Tissot-Dupont, H., Roux, V., Faugere, B., Abegbinni, R. and Birtles, R.J., 1998. Outbreak of epidemic typhus associated with trench fever in Burundi. *The Lancet*, *352*(9125), pp.353-358.

[113] Öztaş, M.O., Önder, M., Öztaş, P. and Atahan, Ç., 2000. Early skin problems after Düzce earthquake. *International journal of dermatology*, *39*(12), pp.952-953.

[114] Alawieh, A., Musharrafieh, U., Jaber, A., Berry, A., Ghosn, N. and Bizri, A.R., 2014. Revisiting leishmaniasis in the time of war: the Syrian conflict and the Lebanese outbreak. *International Journal of Infectious Diseases*, *29*, pp.115-119.

[115] El Mamy, A.B.O., Baba, M.O., Barry, Y., Isselmou, K., Dia, M.L., Hampate, B., Diallo, M.Y., El Kory, M.O.B., Diop, M., Lo, M.M. and Thiongane, Y., 2011. Unexpected rift valley fever outbreak, Northern Mauritania. *Emerging Infectious Diseases*, *17*(10), p.1894.

[116] Reithinger, R., Mohsen, M., Aadil, K., Sidiqi, M., Erasmus, P. and Coleman, P.G., 2003. Anthroponotic cutaneous leishmaniasis, Kabul, Afghanistan. *Emerging Infectious Diseases*, *9*(6), p.727

[117] Dahanayaka, N.J., Kiyohara, T. and Agampodi, S.B., 2013. Massive hepatitis A outbreak in Sri Lanka in 2009: an indication of increasing susceptibility and epidemiological shift?. *Sri Lankan Journal of Infectious Diseases*, *3*(2).

[118] Pal, S., Juyal, D., Sharma, M., Kotian, S., Negi, V. and Sharma, N., 2016. An outbreak of hepatitis A virus among children in a flood rescue camp: a post-disaster catastrophe. *Indian journal of medical microbiology*, *34*(2), p.233.

[119] Boccia, D., Guthmann, J.P., Klovstad, H., Hamid, N., Tatay, M., Ciglenecki, I., Nizou, J.Y., Nicand, E. and Guerin, P.J., 2006. High mortality associated with an outbreak of hepatitis E among displaced persons in Darfur, Sudan. *Clinical infectious diseases*, *42*(12), pp.1679-1684.

[120] Brooks, H.M., Paul, J., Katsuva, M., Claude, K.M., Houston, S. and Hawkes, M.T., 2017. Malaria in an Internally Displaced Persons Camp in the Democratic Republic of the Congo. *Clinical Infectious Diseases*, *65*(3), pp.529-530.

[121] Sugunan, A.P., Roy, S., Murhekar, M.V., Naik, T.N. and Sehgal, S.C., 2007. Outbreak of rotaviral diarrhoea in a relief camp for tsunami victims at Car Nicobar Island, India. *Journal of public health*, *29*(4), pp.449-450.

[122] Yee, E.L., Palacio, H., Atmar, R.L., Shah, U., Kilborn, C., Faul, M., Gavagan, T.E., Feigin, R.D., Versalovic, J., Neill, F.H. and Panlilio, A.L., 2007. Widespread outbreak of norovirus gastroenteritis among evacuees of Hurricane Katrina residing in a large "megashelter" in Houston, Texas: lessons learned for prevention. *Clinical Infectious Diseases*, *44*(8), pp.1032-1039.

[123] Elfaituri, S.S., 2016. Skin diseases among internally displaced Tawerghans living in camps in Benghazi, Libya. *International journal of dermatology*, *55*(9), pp.1000-1004.

[124] Watanabe, H., Batuwanthudawe, R., Thevanesam, V., Kaji, C., Qin, L., Nishikiori, N., Saito, W., Saito, M., Watanabe, K., Oishi, K. and Abeysinghe, N., 2007. Possible prevalence and transmission of acute respiratory tract infections caused by Streptococcus pneumoniae and Haemophilus influenzae among the internally displaced persons in tsunami disaster evacuation camps of Sri Lanka. *Internal Medicine*, *46*(17), pp.1395-1402.
[125] Benny, E., Mesere, K., Pavlin, B.I., Yakam, L., Ford, R., Yoannes, M., Kisa, D., Abdad, M.Y., Menda, L., Greenhill, A.R. and Horwood, P.F., 2014. A large outbreak of shigellosis commencing in an internally displaced population, Papua New Guinea, 2013. *Western Pacific surveillance and response journal: WPSAR*, *5*(3), p.18.
[126] Malholland, K., 1985. Cholera in Sudan: an account of an epidemic in a refugee camp in eastern Sudan, May–June 1985. *Disasters*, *9*(4), pp.247-258.
[127] Liu, L., Haynie, A., Jin, S., Zangeneh, A., Bakota, E., Hornstein, B.D., Beckham, D., Reed, B.C., Kiger, J., McClendon, M. and Perez, E., 2019. Influenza A (H3) outbreak at a Hurricane Harvey Megashelter in Harris County, Texas: Successes and challenges in disease identification and control measure implementation. *Disaster medicine and public health preparedness*, *13*(1), pp.97-101.
[128] Thomson, K., Luis, D.J., Lagu, J., Laku, R., Dineen, B., Schilperoord, M., Muita, M., Gikunju, S., Waiboci, L., Fields, B. and Teshale, E., 2013. Investigation of hepatitis E outbreak among refugees—Upper Nile, South Sudan, 2012–2013. *MMWR. Morbidity and mortality weekly report*, *62*(29), p.581.
[129] Nogodalla, M.A., 2014. Yellow fever outbreak investigation and response, Darfur State, Sudan, September-November 2012. *International Journal of Infectious Diseases*, *21*, p.259.
[130] Prevalence of Cryptosporidium spp. in Children with Diarrhea in the West Bank, Palestine.
[131] Hulland, E., Subaiya, S., Pierre, K., Barthelemy, N., Pierre, J.S., Dismer, A., Juin, S., Fitter, D. and Brunkard, J., 2019. Increase in Reported Cholera Cases in Haiti Following Hurricane Matthew: An Interrupted Time Series Model. *The American journal of tropical medicine and hygiene*, *100*(2), pp.368-373.
[132] Shaman, J., Day, J.F. and Stieglitz, M., 2005. Drought-induced amplification and epidemic transmission of West Nile virus in southern Florida. *Journal of medical entomology*, *42*(2), pp.134-141.

Supplementary Table 1: Full list of included studies in the review

Disaster Type	Disaster	Frequency
**Hydrological**	Flood	43
**Geophysical**	Tsunami	9
	Earthquake	17
	Volcanic eruption	2
**Meteorological**	Cyclone	4
	Typhoon	4
	Tropical Storm	1
	Hurricane	6
**Climatological**	Drought	6
**Conflict**	Armed conflict	45

Supplementary Table 2: Full list of reported disasters

Region	Country	Frequency
**South & South East Asia**	India	12
	Nepal	1
	Bangladesh	4
	Pakistan	1
	Sri Lanka	3
	Sumatra	2
	Philippines	3
	Malaysia	1
	Thailand	2
	Indonesia	2
**Middle East**	Syria	3
	Iraq	2
	Iran	2
	West Bank	1
	Afghanistan	3
	Yemen	4
	Oman	1
	Lebanon	1
**Oceania**	Australia	1
	New Zealand	1
	Fiji	1
	Papua New Guinea	2
	Solomon Islands	1
**Africa**	Libya	1
	Kenya	3
	Ethiopia	2
	Somalia	2
	Sierra Leone	2
	Uganda	2
	Liberia	1
	Mauritania	1
	Mali	1
	Sudan	5
	South Sudan	4
	Central African Republic	3
	Democratic Republic of Congo	2
	Angola	1
	Mozambique	2
	Burundi	1
**East Asia**	China	9
	Japan	4
	Taiwan	2
**Europe**	Bosnia & Herzegovina	2
	Yugoslavia	2
	Czechoslovakia	1
	Croatia	1

	Turkey	2
	Italy	2
	Netherlands	1
	UK	1
	Germany	1
**North America**	USA	10
**Latin America**	Chile	1
	Ecuador	1
	Columbia	1
	Guyana	1
	Mexico	1
	Nicaragua	2
	Costa Rica	1
	Honduras	1
	Haiti	2

Supplementary Table 3: Full list of reported countries

Disease Type	Transmission	Disease	No. of outbreaks
**Bacterial**	Water borne	Leptospirosis	21
	Water borne	Cholera	15
	Water borne	Diarrhoeal disease	7
	Water borne	Dysentery	6
	Water borne	Typhoid	3
	Vector borne	Typhus	2
	Air borne	Pneumonia	2
	Water borne	Bacteremia	1
	Air borne	Diphtheria	1
	Air borne	Meningitis	1
	Air borne	Respiratory disease	1
	Rodent borne	Tularemia	1
**Viral**	Air borne	Measles	5
	Water borne	Hepatitis E	5
	Water borne	Polio	4
	Vector borne	Dengue	3
	Water borne	Diarrhoeal disease	3
	Direct contact	Ebola	2
	Vector borne	Rift Valley fever	2
	Water borne	Hepatitis A	2
	Air borne	Influenza	2
	Vector borne	West Nile	2
	Water borne	Norovirus	2
	Vector borne	Chikungunya	1
	Rodent borne	Hantavirus	1
	Direct contact	Lassa fever	1
	Air borne	Monkeypox	1
	Air borne	Respiratory disease	1
	Air borne	Rubella	1
	Vector borne	Yellow Fever	1
	Vector borne	Zika	1
**Parasitic**	Vector borne	Malaria	10
	Vector borne	Cutaneous leishmaniasis	5
	Water borne	Giardiasis	3
	Vector borne	Visceral leishmaniasis	3
	Water borne	Diarrhoeal disease	2
	Vector borne	Sleeping Sickness	2
	Water borne	Cryptosporidium	1
	Water borne	Fascioliasis	1
	Direct contact	Scabies	1
	Water borne	Schistosomiasis	1
**Mixed pathogen**	Water borne	Diarrhoeal disease	6
	Direct contact	Dermatological disease	3
	Air borne	Respiratory disease	1
		**Total**	140

Supplementary Table 4: Full list of reported disease outbreaks

	p value
**Risk Factor Cluster**	$7.2x10^{-36}$
**Conflict/Displacement**	0.04
**Geophysical/Displacement**	0.22
**Hydrological/WASH**	0.58
**Conflict/WASH**	0.14
**Water-borne/WASH**	0.0022
**Geophysical/Housing**	0.001
**Hydrological/Vector**	0.023
**Conflict/Vector**	0.126
**Parasitic/Vector**	0.004
**Water-borne/Age**	0.14
**Conflict/Age**	0.68
**Conflict/Healthcare**	0.0000003
**Viral/Healthcare**	0.0014
**Hydrological/Gender**	0.004
**Water-borne/Gender**	0.15

Actual p value	Africa	E Asia	Europe	LAC	Middle East	N America	Oceania	S & SE Asia
**Conflict**	0.0000197*		0.79565613	0.04237575 *	0.00000001 *		0.38814874	0.00055075 *
**Hydrological**	0.03742919 *	0.08779559	0.71072159	0.49736414	0.00819448 *	0.29446618	0.31503477	0.04128529 *
**Climatological**	0.00027878 *					0.42591199		
**Geophysical**		0.2537846	0.55776124	0.01565516 *	0.07715278	0.33047572	0.42309951	0.04748551 *
**Meteorological**	0.11744737	0.2876211		0.14102256		0.00488033 *		0.63602109
	**Water-borne**	**Vector-borne**	**Air-borne**	**Rodent-borne**	**Direct contact**			
**Conflict**	0.00002757 *	0.01827592 *	0.32309873	0.04164768 *	0.16000299			
**Hydrological**	0.00000015 *	0.00217725 *	0.0211398*		0.31688118			
**Climatological**	0.16244772	0.11471884			0.18858594			
**Geophysical**	0.00972217 *	0.21803643	0.01387154 *		0.67859545			
**Meteorological**	0.00595018 *	0.10543734	0.52184744					
	**Bacterial**	**Viral**	**Parasitic**	**Mixed pathogen**				
**Conflict**	0.00029837 *	0.00604762 *	0.0463105*	0.11010373				
**Hydrological**	0.00362129 *	0.00795126 *	0.16208116	0.04285716 *				
**Climatological**	0.5726781	0.81973714	0.45566813					
**Geophysical**	0.53167598	0.70071662	0.57786094	0.39523447				
**Meteorological**	0.01736218 *	0.40642793		0.3410774				

Supplementary Table 5: List of full p values from pair-wise comparisons. * shows significance at <0.05. Blank cells in the table indicate no outbreaks that fit both those categories.

Risk Cluster	Risk Factor	No. of outbreaks
**Displacement**		81
A report of national population movement due to the disaster	General	79
	Rural to urban	1
	Coastal to jungle	1
**Age**		40
Reported age-related risk factors, either a demographic group e.g., children, or a specific age category e.g., <5 years.	<2	2
	<4	1
	<5	10
	>5	2
	>5	2
	3-8	1
	<7	1
	<10	1
	Child	2
	<14	1
	15-20	1
	15-19	1
	<15	2
	15-34	1
	<18	2
	<20	2
	40-49	1
	Adults	3
	>50	2
	>65	1
	Older	1
	Elderly	2
**Gender**		25
Males or females being more at risk	Male	20
	Female	5
**WASH**		59
Any issues with access or quality of water, sanitation and hygiene provisions, separate from disruptions to specific municipal services.	Drainage	1
	Sanitation	25
	Hygiene	13
	Drinking water	17
	Toilet/latrine access	3
**Housing**		48
Reports of inadequate living conditions or the location of either habitual residence or temporary housing provided after displacement.	Overcrowding	19
	Urban	1
	Rural	1
	Mountainous	1
	Flooded household	3
	Living a heavily impacted area	6
	Camp-setting	5
	Poor shelter	6
	Sleeping outdoors	2
	Collapsed infrastructure	1
	No bed net	1
	Temporary shelter	2
	Ventilation	1
**Healthcare**		35
	Poor access	12
	Vaccination coverage	11

Any issue that prevented people seeking formal health care	Poor facilities	9
	Willingness/trust to seek care	3
**Municipal services**		14
Disruption to municipal services	Garbage	4
	Waste	6
	Water	4
**Environment**		19
Alterations in the natural environment that exacerbated risk of contracting the disease.	Contact with floodwater	13
	Higher temperatures	2
	Alterations in land moisture	1
	Water salinity	1
	Geological changes	1
	Lower temperatures	1
**Vector/Animal**		38
Changes in animals and vectors that accelerated contact with the population and subsequent disease spread.	Livestock (cattle, pigs, camels)	6
	Rodents	7
	Domestic animals (dogs)	3
	Wildlife (monkeys, bandicoots, beavers)	3
	Alterations in vector breeding ground	14
	Vector control	4
	Vector biting habits	1
	Vector presence	1
	Exposure to animals	1
**Behaviour**		19
Any report of human behaviour which heightened the risk of contact with the pathogen, except human displacement.	Recreation	1
	Swimming	1
	Hiking	1
	Camping	1
	Assisting in clean up	1
	Method of acquiring water and storing	3
	Mixing with people of different immunities	2
	Sexual contact	1
	Not being covered	1
	Sharing combs	1
	Burial practices	2
	Public distrust	1
	Nomadic	1
**Occupation**		13
An occupation which was associated with increased pathogen exposure and therefore disease.	Rice paddy farmer	2
	Street vendor	1
	Unemployed	1
	Homemaker	1
	Farmer	3
	Military	2
	Working outdoors	1
	Hunting	1
	International workers	1
**Nutrition**		12
Issues with insufficient diet or eating specific foods.	No fruits and vegetables	1
	Eating rodents	1
	Eating monkeys	1
	Malnourishment	7
	Drinking sugar cane juice	1
	Eating millet gruel	1
**Co-morbidity**		6
	Pregnancy	2

Significant numbers of infected individuals also presented with another morbidity.	Co-morbidities	2
	Respiratory tract infections	1
	Psychological conditions	1
**Socio-economic**		11
Further socioeconomic conditions that increased cases but did not fit into any other cluster. These mainly included education and inequities.	Poverty	4
	Literacy rate	1
	Poor socio-economics	2
	Education level	2
	Attending church school	1
	Fathers education	1

Supplementary Table 6: Full list of reported risk factors and how they were split into the risk factor clusters. Under each cluster is how they were defined.

Category	Keywords	MeSH MEDLINE	MeSH Embase	MeSH GlobalHealth
**Natural Hazards**	natural hazard* OR natural disaster* OR extreme adj2 event*	climatic process exp, cyclonic storms exp, droughts exp, floods exp, tidal waves exp, geological phenomena exp, avalanches exp, earthquakes exp, landslides exp, tsunamis exp, volcanic eruptions exp, wildfires exp, natural disasters exp	natural disaster exp, disaster victim exp, earthquake exp, drought exp, flooding exp, hurricane exp, tsunami exp, landslide exp, avalanche exp, wildfire exp, volcano exp	natural disaster exp, hurricanes exp, tornados exp, typhoons exp, droughts exp, floods exp, earthquakes exp, landslides exp, avalanches exp, tsunami exp, volcanos exp, wildfire exp
**Conflicts**	armed conflict* or civil war*	ethnic violence exp, exposure to violence exp, armed conflicts exp, war exposure exp	war exposure exp, ethnic conflict exp	conflict exp, war exp, aggression exp, fighting exp
**Disease**	infectious disease outbreak* OR communicable disease outbreak*	disease outbreaks exp, epidemics exp, communicable diseases exp, diarrhoea, vibrio infections exp, cholera exp, salmonella infections exp, typhoid fever exp, paratyphoid fever exp, leptospirosis exp, Weil disease, measles exp, measles virus exp, meningitis, bacterial exp, meningitis, escherichia coli exp, meningitis, meaningococcal exp, meningitis, pneumococcal exp, respiratory tract infections exp, malaria exp, dengue exp, tetanus exp, clostridium infections exp, haemorrhagic fevers, viral exp, poliomyelitis exp, poliovirus exp, coccidioidomycosis exp, dysentery exp, leishmaniasis, cutaneous exp, leishmaniasis, visceral exp, hepatitis exp, hepatitis a exp, hepatitis e exp	typhoid fever exp, salmonellosis exp, acute hepatitis exp, hepatitis a virus exp, hepatitis e virus exp, hepatitis a exp, hepatitis e exp, leptospirosis exp, measles exp, respiratory tract infection exp, malaria exp, dengue exp, tetanus exp, coccidioidomycosis exp, haemorrhagic fever exp, poliomyelitis exp, dysentery exp, acute diarrhoea exp, meningitis exp, skin leishmaniasis exp, visceral leishmaniasis exp	outbreaks exp, epidemics exp, infectious diseases exp, diarrhoea exp, cholera exp, vibrio cholerae exp, salmonella typhi exp, typhoid exp, salmonella paratyphi exp, paratyphoid exp, hepatitis a exp, hepatovirus a exp, hepatitis e exp, hepatovirus e exp, leptospirosis exp, measles exp, meningitis exp, bacterial meningitis exp, viral meningitis exp, respiratory diseases exp, malaria exp, dengue exp, tetanus exp, coccidioidomycosis exp, leishmaniasis exp, cutaneous leishmaniasis exp, visceral leishmaniasis exp, haemorrhagic fever exp, poliomyelitis exp, dysentery exp

Supplementary Table 7: Search strategies for MEDLINE, Embase and GlobalHealth
